# Supplementary material for: Continuous Theta-Burst Stimulation of the Contralesional Primary Motor Cortex for Promotion of Upper Limb Recovery After Stroke: A Randomized Controlled Trial
Source: Stroke. 2023 Jun 22;54(8):1962–71. doi: 10.1161/STROKEAHA.123.042924 (PMC10358447; doi:10.1161/STROKEAHA.123.042924)
Supplement: Supplementary file 1 [file str-54-1962-s001.pdf]

SUPPLEMENTAL MATERIAL

Table S1. *Baseline characteristics*

Patient characteristics	Total (n = 59)	Active cTBS (n = 28)	Sham cTBS (n = 31)
Sex, n (%)			
Male	40 (68)	18 (64)	22 (71)
Female	19 (32)	10 (36)	9 (29)
Age, mean (SD), years	60.2 (12)	56.8 (12)	63.4 (12)
Race, n (%)			
White	53 (90)	24 (86)	29 (94)
Non-white[1]	6 (10)	4 (14)	2 (6)
Education level			
ISCED, median (IQR)	5.0 (3.8)	4.0 (3.0)	6.0 (4.8)
Intervention			
cTBS onset, mean (SD), days post-stroke	14 (4)	14 (5)	15 (4)
Stroke information			
Lesion type, n (%)			
Ischaemic stroke	50 (85)	24 (86)	26 (84)
Intracerebral haemorrhage	9 (15)	4 (14)	5 (16)
Lesion location, n (%)			
Subcortical	24 (41)	12 (43)	12 (39)
Cortical	16 (27)	7 (25)	9 (29)
Brainstem	9 (15)	6 (21)	3 (10)
Unknown	10 (17)	3 (11)	7 (23)
Lesion volume, cm^3 (SD)[2]	27 (49)	25 (40)	29 (56)
Impaired arm, n (%)			
Left arm	33 (56)	15 (54)	18 (58)
Right arm	26 (44)	13 (46)	13 (42)
Dominant arm[3]	24 (41)	15 (56)	9 (29)
Stroke severity on hospital admission			
NIHSS, median (IQR)	7.0 (7.0)	7.0 (7.0)	8.0 (7.0)
Acute intervention			
Intravenous thrombolysis, n (%)	11 (19)	4 (14)	7 (23)
Intra-arterial therapy, n (%)	2 (3)	0 (0)	2 (7)
Both, n (%)	5 (9)	2 (7)	3 (10)
Onset intervention			
Days post-stroke, mean (SD)	16 (4)	16 (4)	17 (4)
Baseline assessment			
Days post-stroke, mean (SD)	14 (4)	14 (5)	15 (4)
Electrophysiology			
MEP presence, n (%)			
Affected hemisphere	20 (34)	11 (39)	9 (29)
Unaffected hemisphere	59 (100)	28 (100)	31 (100)
Resting Motor Threshold, mean (SD), %MO			
Affected hemisphere[4]	84 (26)	84 (23)	83 (28)
Unaffected hemisphere	39 (10)	40 (11)	39 (8)
Baseline function			
Motor impairment, mean (SD)			
Fugl-Meyer Assessment Arm score	25.6 (18.6)	24.1 (18.0)	27.0 (19.3)
Motor function, mean (SD)			

Action Research Arm Test	12.4 (16.6)	11.5 (16.6)	13.2 (16.8)
Motor activity, mean (SD)			
Stroke Upper Limb Capacity Score	3.1 (2.8)	2.9 (2.7)	3.3 (3.0)
Jebsen Taylor Test	100.7 (32.7)	104.3 (28.9)	97.6 (35.8)
Nine Hole Peg Test	1.7 (4.4)	1.3 (3.7)	2.1 (4.9)
Barthel Index	12.4 (4.2)	12.6 (3.9)	12.3 (4.5)
Disability, median (IQR)			
modified Rankin Scale	4 (1)	4 (1)	4 (1)
Quality of life, mean (SD)			
Stroke Impact Scale – upper limb	7.6 (4.3)	7.6 (4.1)	7.6 (4.6)
EuroQol-5D	7.1 (4.3)	6.0 (4.0)	8.2 (4.4)
Other, mean (SD)			
Hospital Anxiety and Depression Scale	10.1 (7.4)	9.9 (5.4)	10.3 (8.9)

[1]Middle-Eastern, Black or Asian. [2]Reported for patients who underwent an MRI scan. [3]Bimanual ignored.[4]RMT was 100%MO if an MEP could not be measured. cTBS: continuous Theta Burst Stimulation; SD: Standard deviation; ISCED: International Standard Classification of Education; IQR: Inter Quartile Range; NIHSS: National Institutes of Health Stroke Scale; MEP: Motor-evoked potential; %MO: Percentage of maximum machine output.

Table S2. Primary and secondary outcomes.

Outcome	Visit (n)	Mean change score (95% CI)		Mean difference in the change score between groups or odds ratio (95% CI)	P-value
		active cTBS	sham cTBS		
**Primary outcome**					
Action Research Arm Test	3 months (56)	27.6 (21.6-33.6)	18.0 (12.3-23.7)	9.6 (1.2-17.9)	0.0244
**Secondary outcomes**					
Motor impairment					
Fugl-Meyer Assessment Arm	<12 hours				
	1 week (58)	17.2 (12.8 to 21.7)	12.2 (7.9 to 16.5)	5.0 (-1.2 to 11.3)	0.1149
	1 month (57)	22.9 (18.1 to 27.7)	15.2 (10.6 to 19.7)	7.7 (1.0 to 14.4)	0.0241
	3 months (55)	24.2 (18.7 to 29.7)	15.1 (9.9 to 20.3)	9.1 (1.5 to 16.7)	0.0196
	6 months (47)	24.6 (18.5 to 30.7)	16.1 (10.4 to 21.8)	8.5 (0.1 to 16.9)	0.0461
	12 months (50)	25.2 (18.1 to 32.3)	17.6 (10.9 to 24.3)	7.6 (-2.3 to 17.5)	0.1316
Motor function					
Action Research Arm Test	<12 hours (59)	15.6 (11.4 to 19.7)	9.2 (5.3 to 13.2)	6.4 (0.6 to 12.1)	0.0310
	1 week (58)	19.1 (14.5 to 23.7)	11.8 (7.4 to 16.2)	7.3 (0.9 to 13.7)	0.0259
	1 month (57)	25.3 (19.9 to 30.7)	16.3 (11.2 to 21.4)	9.0 (1.5 to 16.5)	0.0183
	3 months	See primary outcome			
	6 months (45)	27.5 (20.0 to 35.1)	19.1 (12.1 to 26.2)	8.4 (-2.0 to 18.8)	0.1121
	12 months (49)	31.2 (22.7 to 39.7)	21.4 (13.4 to 29.4)	9.8 (-1.9 to 21.6)	0.1011
Motor activity					
Stroke Upper Limb Capacity Scale	<12 hours				
	1 week				
	1 month (55)	3.6 (2.7 to 4.4)	2.5 (1.7 to 3.3)	1.1 (-0.1 to 2.3)	0.0812
	3 months (55)	4.2 (3.3 to 5.0)	2.9 (2.1 to 3.7)	1.3 (0.1 to 2.5)	0.0496
	6 months (46)	5.0 (4.0 to 6.0)	3.1 (2.2 to 4.0)	1.9 (0.5 to 3.2)	0.0082
	12 months				
Jebsen Taylor Hand Test	<12 hours				
	1 week (57)	-37.9 (-50.1 to -25.7)	-23.4 (-34.8 to -12.0)	-14.5 (-31.4 to 2.4)	0.0911
	1 month (56)	-49.6 (-61.7 to 37.5)	-34.2 (-45.5 to -22.9)	-15.4 (-32.1 to 1.4)	0.0714
	3 months (55)	-58.3 (-71.3 to -45.3)	-39.0 (-51.0 to -26.9)	-19.4 (-37.3 to -1.5)	0.0342
	6 months (45)	-62.7 (-77.7 to -47.6)	-40.8 (-54.5 to -27.0)	-21.9 (-42.5 to -1.3)	0.0372
	12 months				
Nine Hole Peg Test	<12 hours				
	1 week (58)	-2.6 (-5.2 to 0.0)	-2.1 (-4.6 to 0.3)	-0.5 (-4.1 to 3.1)	0.7908
	1 month (57)	-7.1 (-9.7 to 4.6)	-4.0 (-6.4 to -1.6)	-3.1 (-6.7 to 0.5)	0.0873
	3 months (57)	-7.6 (-10.5 to -4.7)	-4.1 (-6.9 to -1.4)	-3.5 (-7.5 to 0.6)	0.0907
	6 months (47)	-10.1 (-13.5 to -6.8)	-4.7 (-7.8 to -1.6)	-5.5 (-10.1 to -0.9)	0.0204
	12 months (49)	-12.8 (-16.5 to -9.1)	-5.1 (-8.6 to -1.7)	-7.6 (-12.7 to -2.5)	0.0036
Barthel Index	<12 hours				
	1 week (58)	6.4 (5.6 to 7.3)	4.7 (3.9 to 5.6)	1.7 (0.5 to 2.9)	0.0069
	1 month (57)	7.1 (6.4 to 7.8)	6.0 (5.4 to 6.7)	1.1 (0.1 to 2.0)	0.0310
	3 months (56)	7.6 (7.1 to 8.2)	6.4 (5.9 to 6.9)	1.2 (0.5 to 2.0)	0.0015
	6 months (57)	7.6 (7.1 to 8.0)	7.1 (6.7 to 7.5)	0.5 (-0.1 to 1.1)	0.1273
	12 months (53)	7.4 (6.9 to 7.9)	7.1 (6.7 to 7.6)	0.2 (-0.4 to 0.9)	0.4564
Disability (OR)					
modified Rankin Scale[1]	<12 hours				
	1 week				
	1 month (57)			0.23 (0.05 to 0.95)	0.0418
	3 months (58)			0.20 (0.05 to 0.79)	0.0225
	6 months (57)			0.39 (0.1 to 1.58)	0.1886
	12 months (53)			1.81 (0.44 to 7.44)	0.4109
Quality of life					

Stroke Impact Scale – upper limb	<12 hours				
	1 week				
	1 month (55)	9.1 (7.2 to 11.0)	6.4 (4.5 to 8.3)	2.7 (-0.1 to 5.4)	0.0552
	3 months (58)	10.4 (8.4 to 12.5)	6.1 (4.1 to 8.1)	4.3 (1.4 to 7.2)	0.0041
	6 months (50)	10.9 (8.7 to 13.1)	7.8 (5.6 to 10.0)	3.1 (-0.1 to 6.2)	0.0545
	12 months				
EuroQol-5D	<12 hours				
	1 week				
	1 month				
	3 months (56)	-4.0 (-5.0 to -3.1)	-3.6 (-4.5 to -2.6)	-0.5 (-1.9 to 0.9)	0.4852
	6 months (55)	-3.8 (-4.8 to -2.8)	-3.9 (-4.9 to -3.0)	0.1 (-1.3 to 1.5)	0.8421
	12 months (51)	-4.6 (-5.5 to -3.6)	-5.1 (-6.0 to -4.2)	0.6 (-0.8 to 1.9)	0.4029

[1]Mean change scores are unavailable because an ordinal statistical analysis was performed. Visits within 12 hours, at 1 week and at 1 month are post-treatment, while visits at 3, 6 and 12 months are post-stroke. n = number of observed data points per visit. OR: Odds ratio.

TEMPLATE RESEARCH PROTOCOL

(October 2015)

- May 2015: adaptation section 11.5: text in accordance to old and new Measure regarding Compulsory Insurance for Clinical Research in Humans
- Sept 2015: adaptation section 9.1, 9.2 and 12.5: text in accordance to WMO amendment on reporting SAE and temporary halt (section 10 of WMO)
- Oct 2015: adaptation section 4.4 – comment [CCMO15], 8.2 and 10.1 with respect to methodology/statistics

PROTOCOL TITLE 'B-STARS: Brain-STimulation for Arm Recovery after Stroke'

**Protocol ID**	***Not applicable***
**Short title**	**B-STARS**
**EudraCT number**	***Not applicable***
**Version**	**9.0**
**Date**	**07-02-2019**
**Coordinating investigator/project leader**	***Prof. dr. Rick M. Dijkhuizen***<br>***Tel.: +31 30 2535569***<br>***E-mail: r.m.dijkhuizen@umcutrecht.nl***
**Principal investigator(s) (in Dutch: hoofdonderzoeker/ uitvoerder)**<br>***<Multicenter research: per site>***<br>***Rehabilitation center De Hoogstraat, Rembrandtkade 10, 3583 TM Utrecht***	***Prof. dr. J.M.A. Visser-Meily***<br>***Universitair Medisch Centrum Utrecht***<br>***Revalidatie, Verplegingswetenschap & Sport***<br>***Heidelberglaan 100***<br>***3584 CX Utrecht***<br>***Huispostnummer W01.121***<br>***Tel: 088 755 88 31***<br>***Tel.: +31 88 7560906***<br>***E-mail: J.M.A.Visser-Meily@umcutrecht.nl***
**Sponsor (in Dutch: verrichter/opdrachtgever)**	***University Medical Center Utrecht***
**Subsidising party**	***Netherlands Organisation for Scientific Research/Netherlands Organisation for Health Research and Development***<br>***Contact:***<br>***ZonMw***<br>***NWO/Innovational Research Incentives Scheme***

	PO Box 93245<br>*2509 AE The Hague*
**Independent expert (s)**	***Dr. V.P.M. Schepers***<br>***Tel.: +31 88 7558831***<br>***E-mail: V.P.M.Schepers-3@umcutrecht.nl***
**Laboratory sites <*if applicable*>**	***Not applicable***
**Pharmacy <*if applicable*>**	***Not applicable***

PROTOCOL SIGNATURE SHEET

**Name**	**Signature**	**Date**
**Sponsor or legal representative:**<br>***<please include name and function>***<br><br>***<For non-commercial research,>***<br>**Head of Department:**<br>**Prof. dr. J.M.A. Visser-Meily**<br>**Head of Research Centre of Excellence in Rehabilitation Medicine Utrecht**		
Principal Investigator<br>***Prof. dr. J.M.A. Visser-Meily***<br>***Professor of Rehabilitation Medicine***<br><br><br>**Coordinating Investigator**<br>***Prof. dr. R.M. Dijkhuizen***<br>***Professor of experimental and translational neuroimaging***		

TABLE OF CONTENTS

1. INTRODUCTION AND RATIONALE 10
2. OBJECTIVES 13
3. STUDY DESIGN 14
4. STUDY POPULATION 16
4.1 Population (base) 16
4.2 Inclusion criteria 16
4.3 Exclusion criteria 16
4.4 Sample size calculation 16
5. TREATMENT OF SUBJECTS 18
5.1 Investigational product/treatment 18
5.2 Use of co-intervention (if applicable) 19
5.3 Escape medication (if applicable) 19
6. METHODS 20
6.1 Study parameters/endpoints 20
6.1.1 Main study parameter/endpoint 20
6.1.2 Secondary study parameters/endpoints (if applicable) 20
6.1.3 Other study parameters (if applicable) 20
6.2 Randomisation, blinding and treatment allocation 21
6.3 Study procedures 21
6.4 Withdrawal of individual subjects 29
6.4.1 Specific criteria for withdrawal (if applicable) 30
6.5 Replacement of individual subjects after withdrawal. 30
6.6 Follow-up of subjects withdrawn from treatment. 30
6.7 Premature termination of the study 30
7. SAFETY REPORTING 31
7.1 Temporary halt for reasons of subject safety 31
7.2 AEs, SAEs and SUSARs 31
7.2.1 Adverse events (AEs) 31
7.2.2 Serious adverse events (SAEs) 31
7.3 Follow-up of adverse events 32
7.4 Data Safety Monitoring Board (DSMB) 32
8. STATISTICAL ANALYSIS 34
8.1 Primary study parameter(s) 34
8.2 Secondary study parameter(s) 34
8.3 Other study parameters 35
8.4 Interim analysis (if applicable) 35
9. ETHICAL CONSIDERATIONS 36
9.1 Regulation statement 36
9.2 Recruitment and consent 36
9.3 Objection by minors or incapacitated subjects (if applicable) 36
9.4 Benefits and risks assessment, group relatedness 36

9.5 Compensation for injury 37
9.6 Incentives (if applicable) 37
10. ADMINISTRATIVE ASPECTS, MONITORING AND PUBLICATION 38
10.1 Handling and storage of data and documents 38
10.2 Monitoring and Quality Assurance 38
10.3 Amendments 38
10.4 Annual progress report 39
10.5 Temporary halt and (prematurely) end of study report 39
10.6 Public disclosure and publication policy 39
11. STRUCTURED RISK ANALYSIS 40
11.1 Potential issues of concern 40
11.2 Synthesis 42
12. REFERENCES 44

LIST OF ABBREVIATIONS AND RELEVANT DEFINITIONS

**ABR**	**ABR form, General Assessment and Registration form, is the application form that is required for submission to the accredited Ethics Committee (In Dutch, ABR = Algemene Beoordeling en Registratie)**
**AE**	**Adverse Event**
**CV**	**Curriculum Vitae**
**DSMB**	**Data Safety Monitoring Board**
**EU**	**European Union**
**GCP**	**Good Clinical Practice**
**IC**	**Informed Consent**
**METC**	**Medical research ethics committee (MREC); in Dutch: medisch ethische toetsing commissie (METC)**
**(S)AE**	**(Serious) Adverse Event**
**Sponsor**	**The sponsor is the party that commissions the organisation or performance of the research, for example a pharmaceutical company, academic hospital, scientific organisation or investigator. A party that provides funding for a study but does not commission it is not regarded as the sponsor, but referred to as a subsidising party.**
**WMO**	**Medical Research Involving Human Subjects Act (in Dutch: Wet Medisch-wetenschappelijk Onderzoek met Mensen**

SUMMARY

Rationale: Many surviving stroke patients are left with moderate to severe functional deficits and long-term dependency on rehabilitation services. The most common functional deficits after stroke are sensorimotor impairments, especially no or limited ability to execute muscle movements with the affected arm or hand.[1-2] Hemiparetic stroke recovery is often associated with an imbalanced interaction between the damaged and undamaged hemispheres, with reduced excitability of the ipsilesional primary motor cortex (M1) while excitability in the contralesional M1 is increased.[19-25] Theta Burst Stimulation (TBS), one of many forms of rTMS, can elicit significant behavioral improvement in recovering stroke patients.[26-28] Despite these promising findings, a randomized controlled trial (RCT) on the long term effects of TBS treatment in subacute, hemiparetic stroke patients is lacking.[35-37] We hypothesize that in stroke patients who receive TBS the upper limb motor recovery is more pronounced (faster with higher motor scores) in contrast to patients receiving sham TBS.

Objective: To determine the therapeutic effect of contralesional cTBS on recovery of function of the paretic arm, at 3 months after ischemic and hemorrhagic stroke. Secondary objectives: 1) To characterize the mode of action of contralesional cTBS on neural network reorganization after ischemic and hemorrhagic stroke, at different time-points, 2) To determine the therapeutic effect of contralesional cTBS on additional sensorimotor functions, at different time-points post treatment, 3) To determine the therapeutic effect of contralesional cTBS on disability and quality of life at different time-points post treatment

Study design: A double-blind randomized placebo-controlled intervention study. Patients will be randomly assigned to one of two groups: one group of patients will receive cTBS stimulation and the other group will receive sham stimulation. Both groups will receive stimulation (followed by standard care upper limb training) for 10 days, during 2 weeks, and will be tested 7 times (in total).

Study population: 60 first-ever unilateral ischemic and hemorrhagic stroke patients with paresis of one arm, defined as a SA score of ≥9 for shoulder abduction (Motricity Index).

Intervention (if applicable): Contralesional cTBS over the hand area of the primary motor cortex on a daily basis for 2 weeks (except the weekends), with a duration of 40 seconds. Sham stimulation will be with the stimulator in sham mode.

Main study parameters/endpoints: Performance on an upper limb function test of the paretic arm.

Nature and extent of the burden and risks associated with participation, benefit and group relatedness: TBS is relatively well-tolerated in the adult and child population according to several systematic reviews. Generally, it is a safe technique, especially when safety guidelines are followed.[58-61] The burden will consist of daily stimulation sessions,

lasting 40 seconds, (during two weeks) and multiple outcome measurements (at baseline and at 6 follow-up time-points). This is extensive, but the research can only be performed with these patient groups (patients in the subacute phase after stroke).

1. INTRODUCTION AND RATIONALE

Stroke – i.e. sudden loss of blood flow to the brain – is the leading cause of adult disability in many countries, including the Netherlands. Worldwide more than 15 million people have a stroke each year. Many surviving stroke patients are left with moderate to severe functional deficits and long-term dependency on rehabilitation services. The burden of stroke will significantly increase with our ageing population. The most common functional deficits after stroke are sensorimotor impairments – e.g. no or limited ability to perceive sensory information and/or execute muscle movements with the affected arm or hand – which occur in the majority of acute stroke patients. Rehabilitation programs contribute to partial functional recovery, and significant improvements in sensorimotor function can be achieved, but most stroke survivors are left with a permanent sensorimotor deficit.[1-2]

Effective therapy for acute stroke is available: thrombolysis or thrombectomy to restore blood flow to the brain.[3, 57] Unfortunately, only 5-15% of stroke patients receive this therapy due to a very narrow treatment time-window.[4] Hence, there is urgent need for additional therapeutic strategies that are beneficial and safe when applied at later stages, as emphasized in several recent review papers.[5-9] Recent studies suggest that functional improvement after stroke may be augmented by a promising strategy that involves neuromodulation through non-invasive brain stimulation, such as transcranial magnetic stimulation (TMS), in combination with rehabilitative training.[10-15] TMS provides a non-invasive and safe way to directly facilitate or suppress brain activity to modulate motor functions. TMS induces current in the cortex with a coil that generates a magnetic field.[16] Delivery of repetitive trains of TMS (rTMS) at high-frequency enhances cortical excitability, while repetitive low-frequency TMS suppresses cortical excitability.[16] Recently, patterned protocols consisting of short trains of high-frequency TMS (30-100 Hz) in the theta-frequency range (4-7 Hz) (theta-burst stimulation (TBS))[17] have been shown to provide effective and reliable paradigms for excitatory (intermittent TBS (iTBS)) or inhibitory (continuous TBS (cTBS)) brain stimulation, with lasting effects that exceed those induced by standard rTMS protocols.[18]

Hemiparetic stroke patients often have a functionally imbalanced interaction between the damaged and undamaged brain hemispheres, with reduced excitability of the ipsilesional primary motor cortex (M1) while excitability in the contralesional M1 is increased.[19-25] Recent proof-of-principle studies have demonstrated that specific TMS paradigms – i.e. facilitatory stimulation of the affected hemisphere to upregulate excitability, or inhibitory stimulation of the unaffected hemisphere to downregulate excitability – can elicit significant behavioral improvement in recovering stroke patients.[26-28] Most of these studies applied single stimulation sessions, but data suggest that longer lasting effects on motor function, up to at least a year after treatment, can be achieved by multiple sessions of rTMS in acute/subacute

stroke patients.[29-30] This probably reflects long-term potentiation (LTP)- or long-term depression (LTD)-like cortical plasticity, induced by facilitatory or inhibitory stimulation, respectively.[31-33] As non-invasive brain stimulation can prime M1, additional rehabilitative training may further enhance the beneficial effects of TMS.[15, 34] TBS paradigms are particularly promising because sessions are shorter (i.e. more practical), and effects are longer-lasting (i.e. more effective) as compared to standard rTMS protocols. The feasibility, safety and (partial) efficacy of TBS in hemiparetic stroke patients has been demonstrated in a number of studies.[34-37] cTBS of the intact contralesional M1 offers the most straightforward and accurate approach, as this region is easily identified from single-pulse TMS-induced motor evoked potentials (MEPs), which is more complicated in the structurally and/or functionally injured ipsilesional M1.[38-39] In a recent study with a small group of chronic hemiparetic stroke patients (n=10), daily cTBS sessions combined with occupational therapy led to significant improvement in motor function of the upper limb after a 15-day protocol.[40]

Despite these promising findings, a randomized controlled trial (RCT) on the long-term effects of TBS treatment in hemiparetic stroke patients is lacking. Moreover, most earlier studies involved chronic patients in whom post-stroke neural network reorganization had probably stabilized already, which may have limited the therapeutic potential of TBS.[34-37] A RCT in subacute stroke patients would provide important new insights on the therapeutic efficacy of this non-invasive and practicable intervention during an optimal time-window for neurorehabilitation after stroke, especially in combination with a straightforward upper limb training approach, such as exercises described in the recently developed Exercise Guide.[80] In 2010 this guide was developed for the project 'Snel in Beweging' by the University Medical Center Utrecht and Rehabilitation Center De Hoogstraat in collaboration with seven hospitals, rehabilitation centers and nursing homes, and patients of the Dutch stroke patients association. The guide was translated into English in 2013. The core idea is the importance of the intensity of the rehabilitation treatment after stroke: the more therapy, the more recovery during the first 6 months after stroke.[81-83] Patients can start exercising independently from day one after stroke. The application of the exercise guide has been shown to be effective, because the implementation of the guide, together with some other interventions, increased the time spent on moderate to intensive activities (24% versus 38% before and after the implementation) and patients practiced more independently and under supervision (3% versus 6% before and after the intervention).[84]

In addition to lack of knowledge on the extent of cTBS-induced effects on post-stroke functional recovery, we don't know the underlying mode of action of cTBS – specifically its influence on neural network reorganization over prolonged periods. Neither do we know how to efficiently optimize or monitor the effects of therapeutic cTBS. Hypothetically, suppression of hyperexcitability in contralesional M1 would relieve transcallosal inhibition of ipsilesional

M1, particularly subacutely after stroke when interhemispheric functional connectivity is disturbed and contralesional hyperactivation is associated with poor outcome.[41-42] These effects may be ideally measured with MRI, which enables non-invasive assessment of brain structure and function over time. We and others have shown with functional MRI (fMRI) that preservation or reinstatement of perilesional activity is strongly associated with functional recovery after stroke.[43-44] Furthermore, interhemispheric interactions may be critically involved in this process, as recently demonstrated with resting-state fMRI.[45-46] In addition, with diffusion tensor imaging (DTI), an MRI method for the assessment of neuroanatomical structure, we found that 1) a preserved corticospinal tract is predictive of good motor outcome, and 2) sensorimotor network rearrangements are accompanied by improvement of structural integrity in neuronal tract regions.[47] Thus, MRI offers a powerful tool to monitor the effects of non-invasive brain stimulation on structural and functional neural networks, and to identify biomarkers that can predict to what extent a patient will be able to recover and/or benefit from this therapy. Finally, the views and experiences from stroke patients who have undergone the TBS treatment can best be recorded through an interview. Combining quantitative and qualitative data will give a broader picture of the feasibility of this type of intervention.

2. OBJECTIVES

Primary Objective:
To determine the therapeutic effect of contralesional cTBS on recovery of function of the paretic arm, at 3 months after ischemic and hemorrhagic *stroke*

Secondary Objectives:
To characterize the mode of action of contralesional cTBS on neural network reorganization after ischemic and hemorrhagic *stroke, at different time-points*

To determine the therapeutic effect of contralesional cTBS on additional sensorimotor functions, at different time-points post treatment

To determine the therapeutic effect of contralesional cTBS on disability and quality of life at different time-points post treatment

To explore the experiences of stroke patients participating in a clinical trial for upper limb recovery following cTBS

3. STUDY DESIGN

The study will have a RCT design. Subjects will be randomly allocated to real or sham stimulation, and blinded for the specific treatment. Non-invasive brain stimulation will involve daily sessions of cTBS of the contralesional hand area of the primary motor cortex over a period of two weeks (5 days a week). Duration of the cTBS is only 40 seconds. cTBS will be directly followed by upper limb training, part of standard care in rehabilitation program in De Hoogstraat. However, for this study, the upper limb training is now linked to the cTBS. The patients will be recruited from the University Medical Center Utrecht and rehabilitation center de Hoogstraat over a period of 3 years. Only patients who are going to revalidation center De Hoogstraat for their revalidation process, are eligible for the study. Follow-up will continue until one year after inclusion of the last patient. The total study period is expected to be four years. *Figure 1* gives a schematic overview of the study procedures.

Sensorimotor function testing and diagnostic TMS will be conducted one to three days before brain stimulation, one week (±2 days) and one month (±4 days) after stimulation and 90 days (±14 days), 180 days (±14 days) and one year (±14 days) after stroke onset. The primary outcome measure will be assessed on the last day of stimulation as well. Primary outcome measure will be the Action Research Arm Test (ARAT) score at 3 months post stroke, which assesses the ability to perform gross movements and the ability to grasp, move and release objects differing in size, weight and shape.[48] The ARAT will be applied similarly as described in the EXPLICIT program, which also has taken place at the UMCU and rehabilitation center De Hoogstraat.[53] Additional sensorimotor function tests will include the Fugl-Meyer upper extremity (FM-UE) score, Nine Hole Peg Test (9HPT), Jebson-Taylor hand test (JTT), Stroke Upper Limb Capacity Scale (SULCS), skilled reaching and Finger Tapping (FT). Other questionnaires will include the Hospital Anxiety and Depression Scale (HADS), modified Rankin Scale (mRS), Stroke Impact Scale (SIS) and EuroQol-5D (EQ-5D). Corticospinal excitability and intracortical inhibition will be assessed from MEP responses induced by single-pulse TMS to the ipsi- and contralesional M1, measured by EMG of the first dorsal interosseous (FDI) muscle of both hands.[18]

Patients will undergo MRI (optional) to measure ischemic and hemorrhagic injury (structural MRI), white matter integrity (diffusion tensor imaging), functional connectivity (resting-state fMRI) and cortical activation (task-related fMRI) prior to brain stimulation, and one week, three months, six months and one year after stimulation. Optionally, patients can share their views and experiences about the cTBS treatment in a single interview.

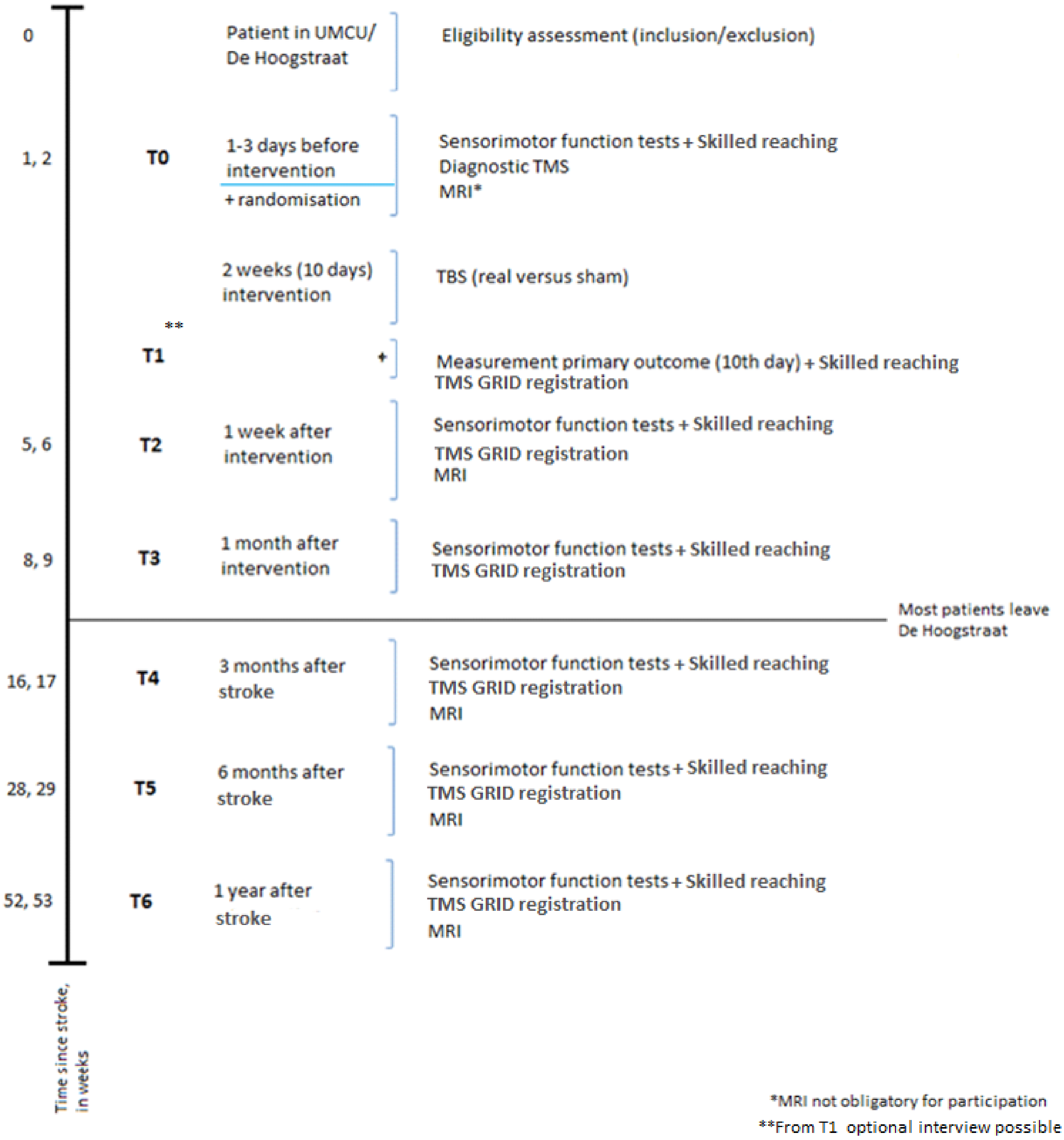

Figure 1. Schematic overview of study procedure

4. STUDY POPULATION

4.1 Population (base)

Recruitment of the participants will be performed in the University Medical Center Utrecht (UMCU) and rehabilitation center De Hoogstraat. The study population will consist of 60 patients within 21 days of stroke onset, in whom there is an optimal time window for rehabilitative treatment because of enhanced plasticity in the brain.

4.2 Inclusion criteria

In order to be eligible to participate in this study, a subject must meet all of the following criteria:

1) Adult patient age ≥ 18;
2) first-ever unilateral ischemic and hemorrhagic stroke (i.e. within cerebral hemispheres, brainstem);
3) paresis of one arm, with a SA score shoulder abduction ≥9 (Motricity Index)
4) within the first 3 weeks after stroke onset;
5) signed informed consent.

4.3 Exclusion criteria

A potential subject who meets any of the following criteria will be excluded from participation in this study:

1) Disabling medical history (severe or recent heart disease, severe head trauma, coercively treated at a psychiatric ward);
2) history of epilepsy;
3) normal to almost normal use of hand; maximum Motricity Index hand score of 33
4) severe deficits in communication, memory, or understanding that impede proper study participation, as determined by the treating physician;
5) contraindications for TMS and MRI[18, 51] (e.g. metal (implants) in skull/scalp/head or fragments from welding or metalwork, implanted device, pregnancy). N.B. metal fillings (i.e. conductive) or non-ferromagnetic dental implants are an exception to the rule.

4.4 Sample size calculation

Sample size will be 60 patients, 30 patients in each group, based on a recent meta-analysis that showed a mean effect size on motor outcome after rTMS of 0.55 with a confidence interval 0.18 at a confidence level of 95% (calculated at www.surveysystem.com/sscalc.htm).[62]

Analysis is based on a statistical power of 80%, alpha of 5% and an effect size of 0.55. The statistical program G*Power has been used for this analysis.[98]

**Analysis:**	A priori: Compute required sample size		
**Input:**	Effect size f(V)	=	0.55
	α err prob	=	0.05
	Power (1-β err prob)	=	0.80
	Number of groups	=	2
	Number of measurements	=	7
	Nonsphericity correction ε	=	0.75
**Output:**	Noncentrality parameter λ	=	12.7050000
	Critical F	=	2.3232187
	Numerator df	=	4.5000000
	Denominator df	=	243
	Total sample size	=	56
	Actual power	=	0.8016012

As additional support for our sample size, we refer to Suppa et al. (2016) who have suggested that an amount of 30 people per group is sufficient to reliably detect a difference in response magnitude of at least 20%.

5. TREATMENT OF SUBJECTS

5.1 Investigational product/treatment

All patients will receive standard rehabilitation therapy. In addition, real cTBS or sham cTBS in combination with upper limb training will be applied in daily sessions during two weeks (10 working days), starting within 21 days after stroke onset. cTBS will only be performed at rehabilitation center De Hoogstraat. The stimulation will be performed by the researchers with the necessary knowledge and skills. We will employ a standard cTBS paradigm consisting of three stimuli bursts at 50 Hz, repeated at 5 Hz frequency, resulting in 600 stimuli in 40 seconds.[17] cTBS intensity will be at 70% of resting motor threshold, which induces highly consistent LTD-like MEP depression with low intersubject variability.[49] Sham stimulation will be done with the stimulator in sham mode. We will use a figure-of-eight coil and the magnetic stimulator 'Neuro-MS', which will be purchased for this research (CE-certificate). These coils are tailor-made to provide therapeutic rTMS and placebo stimulation without overheating.[50] For each session, resting motor threshold will be determined from EMG (recorded with two Ag/AgCl surface electrodes) from the contralateral FDI muscle. A digital neurophysiological system for EMG will be used (CE-certificate).The motor threshold will be defined as the minimum intensity of TMS over the hand area of the contralesional primary motor cortex to elicit at least five contralateral MEPs with >50 µV peak-to-peak amplitude in ten trials with 7 s intertrial intervals. After the first treatment session the RMT will be checked again for the stimulated hemisphere. The optimal position to evoke a MEP from contralateral FDI will be guided by the Neural Navigator (if the CT scan from the standard care has a good quality or when the MRI scan is made before stimulation) to ensure consistent coil placement for cTBS (CE-certificate, Brain Science Tools BV). Applied protocol(s) will be in accordance with most recent safety and tolerability guidelines for TMS applications.[18, 51-52, 85]

Directly after each cTBS session, subjects will undergo upper limb training as part of standard care. This upper limb training consists amongst others of exercises from the Exercise Guide.[80] In 2010 this guide was developed for the project 'Snel in Beweging' by the University Medical Center Utrecht and Rehabilitation Center De Hoogstraat in collaboration with seven hospitals, rehabilitation centers and nursing homes, and patients of the Dutch stroke patients association. The guide was translated into English in 2013. There are 3 different levels depending on the experienced problems by the patients. The selection of exercises will be done by the treating therapist (ergotherapist and/or physical therapist).

5.2 Use of co-intervention (if applicable)

Patients in both treatment groups will be treated according to the standard care in rehabilitation center De Hoogstraat. Patients are divided into 6 subgroups (1a, 1b et cetera) depending on their motor abilities, cognitive abilities and the presence of aphasia. Placement into one of the 6 groups determines the weekly program with physiotherapy, occupational therapy, hand group, speech therapy et cetera.

5.3 Escape medication (if applicable)

Not applicable.

6. METHODS

6.1 Study parameters/endpoints

6.1.1 Main study parameter/endpoint

The primary functional outcome measure will be the change in ARAT score as described by Van der Lee et al.[54] assessed at 3 months post stroke.

6.1.2 Secondary study parameters/endpoints (if applicable)

In addition to the ARAT score, we will measure sensorimotor function with the following tests: Fugl-Meyer score, Nine Hole Peg Test, Jebson-Taylor hand test, Stroke Upper Limb Capacity Scale, skilled reaching and Finger Tapping.

The following outcome measurements will be used to measure dependency, quality of life and depression: modified Rankin Scale, Stroke Impact Scale, Hospital Anxiety and Depression Scale and EQ-5D.

The characteristics of motor-evoked potentials evoked by single pulse TMS will be assessed. Single pulse TMS will be delivered to several targets in a grid which is projected onto the left and right primary motor cortices at T0-T6.

To assess patients' functional and structural brain status, we will measure fMRI-based sensorimotor activation (i.e. amount of voxels with significant task-induced responses), resting-state fMRI-based neuronal network parameters (i.e. functional connectivity between regions) and DTI-based white matter integrity (i.e. fractional anisotropy) within the bilateral sensorimotor system.

These secondary parameters will be measured at baseline, 1 week (±2 days), 1 month (±4 days), 90 days (±14 days), 180 days (±14 days) and 1 year (±14 days) after stimulation.

To explore the views and experiences of the included patients in the trial, a single interview will be held, when the last treatment session has been performed. Open questions will be asked to allow patients to express their views on the brain stimulation intervention.

6.1.3 Other study parameters (if applicable)

The following parameters will be used to control for possible interfering effects (based on information from the medical records):

- Demographic parameters: gender, age, education level, handedness, marital status, ethnicity

- Stroke related parameters: type of stroke, stroke severity (NIHSS, BI), side affected limb, days since stroke onset, cognition (MOCA)

These parameters will only be assessed at baseline (they are not expected to be influenced by the cTBS intervention).

6.2 Randomisation, blinding and treatment allocation

Patients will be stratified based on the severity of their arm paresis. Based on the EXCITE study[101] the patients will be divided into 2 groups (higher- and lower functioning), and then randomly assigned to either the real cTBS or sham cTBS group. A randomisation list will be used. Randomisation will be performed as late as possible, after the baseline assessment, because of possible improvements in motor function during the first days after stroke. Based on some set criteria patients will be divided into a higher- and lower functioning group.[101] Higher functioning patients must demonstrate a minimal presence of finger extension; thumb or one or more fingers that can move somewhat arbitrarily. Lower functioning patients must demonstrate no voluntary movement in fingers.[101]

Randomisation will take place at the Julius data management department through a computerized method. The randomisation code is kept at the Julius data management department. Prof. dr. Visser-Meily has access to the randomisation code when needed. This may occur during a research visit when according to the physician the symptoms of the patient are such that the code needs to be broken.

The investigator will execute the treatment. The main study parameter, the ARAT score, will be measured by other trained and blinded researchers not involved in the research (at the 3 month time-point). The other tests will be tested by the investigator. The nurses, physicians, and physical therapist(s) who will observe the patients until the final study time-point will be unaware of the patient's group assignment.

6.3 Study procedures

Patients will be recruited and included from the UMC Utrecht (only patients who are moving to De Hoogstraat for their revalidation process) and rehabilitation center De Hoogstraat. Patients who fulfil the study criteria will be asked to participate, and they will receive a Patient Information Letter explaining the background and methods of the study. Patients can decide to participate with or without additional MRI scanning at the UMC Utrecht. After written informed consent, patients will be randomly

allocated to the treatment procedures. A TMS questionnaire to confirm safe participation is collected. The first assessment of outcome parameters will take place in the first 21 days after stroke onset, which includes sensorimotor function tests and diagnostic TMS. Patients who approve a MRI scan, will also undergo MRI at this stage. One to three days after the first assessment, patients will undergo daily treatment sessions (with a duration of 40 seconds) of cTBS or sham cTBS (10 working days) in combination with upper limb training during two weeks, at the rehabilitation center De Hoogstraat. Patients not involved in the research and patients receiving sham stimulation will receive the upper limb training as well, because it's part of the standard care in the rehabilitation center. Subsequently, sensorimotor function testing and diagnostic TMS will be done at one week, one month, three months, six months and one year after the cTBS or sham cTBS treatment. The primary outcome measure will be tested at the last stimulation day. Additional MRI will be executed after the first week, the third month, the sixth month and one year after real/sham cTBS treatment. The optional interview will be scheduled when the last treatment session has been performed. A schedule of all the assessments can be found in Table 1-3. Table 1 is an overview of all the sensorimotor function tests and questionnaires. Table 2 provides information about all the single-TMS and (f)MRI measurements. All patients will receive standard care and rehabilitation therapy. Table 3 is an overview of all the measurements that are part of standard care.

	Instrument	T0	T1	T2	T3	T4	T5	T6	Assessment time
**Primary Outcome measure**									
Motor function	Action Research Arm Test (ARAT)	X	X	X	X	X	X	X	max. 20 min
**Secondary outcome measures**									
**Function**									
Motor function	Fugl-Meyer (FM)	X		X	X	X	X	X	max. 20 min
Motor function	Stroke Upper Limb Capacity Scale (SULCS)	X			X	X	X		max. 6 min
Motor function	Finger Tapping (FT)	X			X	X	X	X	max. 1 min
Motor function	Skilled reaching	X	X	X	X	X	X	X	max. 4 min
**Activities**									
Coordination	Nine-hole Peg Test (9HPT)	X		X	X	X	X	X	max. 10 min
Speed	Jebsen Taylor Test (JTT)	X		X	X	X	X		max. 10 min
**Participation**									
Quality of life	Stroke Impact Scale (SIS; hand function subscale + thermometer	X			X	X	X		max. 3 min
	EuroQol-5D (EQ-5D)	X				X	X	X	
Dependency	Modified Rankin Scale	X			X	X	X	X	max. 2 min
**Determinants/ characteristics**									
**Stroke related factors**									
Mood	Hospital Anxiety and Depression Scale (HADS)	X			X	X	X		2-6 min

Table 1. Overview of all measures (motor function tests and questionnaires) for the stroke patient and the moment of administering. The first assessment (T0) takes place in the first 7-14 days post-stroke. The follow-up assessments are at the last day of stimulation session (T1), 1 week (T2), 1 month (T3) after stimulation, 3 months (T4), six months (T5) and 1 year (T6) post-stroke.

	Instrument	T0	T1	T2	T3	T4	T5	T6	Assessment time
**Secondary outcome measures**									
**Brain reorganization**									
Corticospinal excitability and intracortical inhibition	Single-pulse TMS	X	X	X	X	X	X	X	15 min
Ischemic/hemorrhagic injury, white matter integrity, functional connectivity, and cortical activation	(f)MRI (optional)	X		X		X	X	X	25 min
**Qualitative study**									
Personal views and experiences from patients	Interview			X					30-60 min

Table 2. Overview of all measures for the stroke patient and the moment of administering. The first assessment (T0) takes place in the first 7-14 days post-stroke. The follow-up assessments are at the last day of stimulation session (T1), 1 week (T2), 1 month (T3) after stimulation, 3 months (T4), six months (T5) and 1 year (T6) post-stroke.

	Instrument	T0	T1	T2	T3	T4	T5	T6	Assessment time
**Secondary outcome measures**									
**Activities**									
Activities of Daily Living (ADL)	Barthel Index	X		X*	X*	X*	X*	X*	2-5 min *= not standard care
**Determinants/characteristics**									
Demographics	Age, gender, education, marital status, ethnicity, work status, handedness, medication	X							-
**Stroke related factors**									
Cognition	Montreal Cognitive Assessment (MOCA)	X							max. 10 min
Stroke characteristics	Type of stroke, stroke severity (NIHSS, MI), side affected limb	X							-

Table 3. Overview of all measures part of care as usual. The first assessment (T0) takes place in the first 7-14 days post-stroke. The follow-up assessments are at the last day of stimulation session (T1), 1 week (T2), 1 month (T3) after stimulation, 3 months (T4), six months (T5) and 1 year (T6) post-stroke.

Sensorimotor function tests

ARAT

The ARAT is a performance test which assesses the ability to perform gross movements and the ability to grasp, move and release objects differing in size, weight and shape (F1). The original test consists of 19 items, rated on 4-point ordinal scales (0 to 3), with a maximum score of 57. The better the patient, the higher the score. By removing four items, a hierarchical 1-dimensional scale has been constructed.[54]

Fugl-Meyer

The Fugl-Meyer arm score is a reliable and valid motor performance test consisting of 33 tasks performed by the affected upper limb .[64-65] (F1). The FM-arm test evaluates the ability to make movements outside the synergistic pattern. Performance on each task is rated as 0, 1 or 2, with higher ratings representing better performance. The FM-arm measure will be used as the sum of 33 ratings (possible range 0 to 66 points). The Fugl-Meyer assessment also has a lower extremity part. These tasks are also rated on a 0-2 point scale with a maximum of 34 points. This section of the assessment will also be measured to see if the stimulation has effects on the lower extremities.

Nine Hole Peg Test

The Nine Hole Peg Test (9HPT) examines the speed of movement of amongst others the fine motor skills (F1). The patient has to grab as quickly as possible 9 pegs from a bowl and put these into the openings of nine holes. Afterwards, the patient has to take the 9 pegs out of the holes again and put them back in the bowl. The duration of this operation is measured. The maximum time is 50 seconds, and then the number of pegs is counted. You can earn one point by place the peg into a hole, as well as take the peg out of the hole and put them back into the bowl. So you can achieve a maximum of 18 points. The patient can only use the affected hand and must take the pegs one by one.[63] Reliability and validity have been demonstrated in patients with stroke.[66]

Jebsen-Taylor hand test

Hand skill is measured by the Jebsen-Taylor hand test (F1). A total of 7 subtests have to be performed, picking up small objects and place them in a container, card turning, stimulated feeding et cetera. Each item is scored according to time taken to complete the task. The scores for all 7 items are then summed for a total score.These hand functions refer to activities of daily living.[74] The tasks have to be executed first with

the non-dominant hand, and then with the dominant hand. Hereby it is possible to see the effects of the stimulation on the unaffected hand.

Stroke Upper Limb Cacapity Scale

The SULCS consists of 10 items, each of which may receive a score of 0 or 1 (maximum score of 10; F1). Score of 1 on an item means that the patient is able to perform the task in the manner described. The items are focused on arm capacity, and basal and complex hand capacity. The items have an hierarchical order. Two scientific papers showed excellent clinimetric properties of the SULCS.[99,100]

Skilled reaching

The skilled reaching task assesses skilled reaching behavior and can be scored on multiple components (success score, first attempt, movement elements).
Patientss will be seated in an armless chair, feet flat on the ground and their hands palm down on their thighs with the fingers extended. A small food item (Honey Loops, smarties, raisin, shelled peanut) will be placed on a pedestal placed in front of them, adjusted to the trunks' height and arm length. Each hand will be used to make three to five reaches, accomplished within a few minutes (Klein, 2009; Melvin et al., 2005; Whishaw et al., 2002). The patients will have to reach for the food item and withdraw this to their mouth. The tasks will be video-recorded with a camera with a shutter speed of 1/1000 frames per second, to enable frame-by-frame analysis.

Finger Tapping

Finger tapping is an index of skilled movement or movement speed. Tapping frequency is measured by tapping with the index finger as fast as possible during 30 seconds. The total number of hits is counted.[105]

<u>Brain reorganization (TMS and MRI)</u>

Motor-evoked potentials (MEPs) evoked by single pulse TMS to the motor hotspot in the ipsi- and contralesional hemisphere will be assessed at T0-T6. Single pulse TMS will be delivered to several targets in a grid which is virtually projected onto the left and right primary motor cortices to visualize the changes in cortical motor representation. During the grid registration, EMG will be measured in the bilateral first dorsal interosseous, contralesional anterior pollicis brevis and contralesional abductor digiti minimi muscles. The grid is centered at the maximum MEP amplitude (as determined during the resting motor threshold procedure) and consists of 4 by 4 targets with 8 mm spacing between the targets. 5 repetitions of single pulse TMS will be delivered at 120% relative to the resting motor

threshold to each target. In total, 125 pulses will be delivered. A beta version of the neural navigator software will be used to register the TMS coil position at the moment of TMS pulse delivery and visualization of this location on the brain surface.

Additionally, 5 TMS pulses will be delivered to 7th cervical vertebra in the neck to obtain the peripheral conduction time. The peripheral conduction time will be used to correct latencies for differences in upper limb length and differences in peripheral conduction velocities. This procedure is also applied clinically in clinical neurophysiological exams and is experienced similarly compared to cortical stimulation.

MRI will be executed on a clinical 3T scanner. The MRI protocol will include standard anatomical MRI (4-8 minutes), DTI (6 minutes), task fMRI (5 minutes) and resting-state fMRI (6 minutes), during which the patient is requested to lie still and relax with eyes closed. Task-related fMRI will be done during flexion-extension movement in a blocked design, comparable to the fMRI task described by Buma et al. (2015)[103]. Before fMRI scanning, patients will be trained to perform the task correctly. The patients will wear a data glove on each hand. Both arms will rest comfortably in a suspine position with the elbows bent in a comfortable position for the patient. The task will be presented on a screen. On the screen there are two hands moving up and down in a vertical fashion (representing the extension of the fingers and bending of the fingers in 90° flexion). The patients will be asked to follow the hand to the best of their ability. Movement of the individual left hand and right hand is alternated with rest.

<u>Other measures</u>

Barthel Index (BI)

The BI is an ordinal scale used to measure performance in 10 activities of daily living (F1). Test scores range from 0 to 100, with higher scores indicating better performance in these activities.[75-77]

Modified Rankin Scale

The Modified Rankin Scale is an observation list to determine the functional status of a person post stroke (F1). The functional status of patients can be subdivided into 6 subscales. Score '0' corresponds to no symptoms and score ‘5’ corresponds to severe handicap.[73]

Stroke Impact Scale

The Stroke Impact Scale (SIS) is a self-report health status measure, specifically for the stroke population (F1). This multidimensional instrument measures hand function,

strength, activities of daily living, communication, emotion, memory and thinking.[78] The hand item (question 7) and the last question about the subjective recovery after stroke (question 9) will be used.

Hospital Anxiety and Depression Scale

The Hospital Anxiety and Depression Scale (HADS) measures the core symptoms of anxiety and depression without involving physical symptoms (F1). It is an easy to use, short questionnaire. It consists of a depression scale and an anxiety scale, each containing 7 items.[79] The highest possible score is 21, scores between 11-21 are indicative for a probable depression.

EuroQol-6D

The EuroQol-5D is a very short, efficient and general questionnaire. It consists of 5 questions with each 5 answer possibilities. Each question captures one dimension of quality of life; mobility, self-care, daily activities, pain or other complaints and anxiety/depression. We added a sixth question in the theme of cognition based on the EQ-6D, but with 5 answer possibilities instead of 3. The result of this questionnaire is a 5-digit number than can be converted into a preference weight which is also called a single weighted index score. The EQ-5D has a good validity and is an efficient tool to measure health status.[104]

Interview

Open questions will be asked to allow patients to express their views on the brain stimulation intervention.

6.4 Withdrawal of individual subjects

Subjects can leave the study at any time for any reason if they wish to do so without any consequences. The investigator can decide to withdraw a subject from the study for urgent medical reasons. The collected questionnaires and cognitive data will be destroyed on request of the withdrawn participant.

6.4.1 Specific criteria for withdrawal (if applicable)

Not applicable

6.5 Replacement of individual subjects after withdrawal

There will be no replacement of individual participants that withdraw.

6.6 Follow-up of subjects withdrawn from treatment

Patients who withdrew from this study will receive a follow-up measurement, when there is permission.

6.7 Premature termination of the study

This study is under surveillance of a Data Safety Monitoring Board, which can advise the sponsor to terminate the study prematurely; see 7.4.

7. SAFETY REPORTING

7.1 Temporary halt for reasons of subject safety

In accordance to section 10, subsection 4, of the WMO, the sponsor will suspend the study if there is sufficient ground that continuation of the study will jeopardise subject health or safety. The sponsor will notify the accredited METC without undue delay of a temporary halt including the reason for such an action. The study will be suspended pending a further positive decision by the accredited METC. The investigator will take care that all subjects are kept informed.

7.2 AEs, SAEs and SUSARs

7.2.1 Adverse events (AEs)

Adverse events are defined as any undesirable experience occurring to a subject during the study, whether or not considered related to cTBS in combination with upper limb training. All adverse events reported spontaneously by the subject to (or observed by) the investigator or his staff will be recorded for the period of the stimulation (2 weeks) and an additional week after the stimulation has stopped. Furthermore, all adverse events occurring within 24 hours after MRI will be reported as well. During the follow-up period (only diagnostic measurements) only SAEs will be reported in the annual progress report, see below.

7.2.2 Serious adverse events (SAEs)

A serious adverse event is any untoward medical occurrence or effect that

- results in death;
- is life threatening (at the time of the event);
- requires hospitalisation or prolongation of existing inpatients' hospitalisation;
- results in persistent or significant disability or incapacity;
- is a congenital anomaly or birth defect; or
- any other important medical event that did not result in any of the outcomes listed above due to medical or surgical intervention but could have been based upon appropriate judgement by the investigator.

An elective hospital admission will not be considered as a serious adverse event.

The sponsor will report the SAEs (AEs which develop into SAEs), occurring during the 3-week period from the start of the stimulation, through the web portal

ToetsingOnline to the accredited METC that approved the protocol, within 7 days of first knowledge for SAEs that result in death or are life threatening followed by a period of maximum of 8 days to complete the initial preliminary report. All other SAEs will be reported within a period of maximum 15 days after the sponsor has first knowledge of the serious adverse events. SAEs occurring within 24 hours after MRI will be reported. SAEs occurring during the follow-up period will be reported in the annual progress report. Stroke survivors are often plagued by medical problems, and side effects of brain stimulation will only occur during and in rare cases for a short time period after brain stimulation.

7.3 Follow-up of adverse events

All AEs will be followed until they have abated, or until a stable situation has been reached. Depending on the event, follow up may require additional tests or medical procedures as indicated, and/or referral to the general physician or a medical specialist. SAEs need to be reported till end of study within the Netherlands, as defined in the protocol

7.4 Data Safety Monitoring Board (DSMB)

The central, internal DSMB of the UMC Utrecht is established to perform ongoing safety surveillance. The current composition of the internal DSMB is:

· Prof. dr. M.J.C. Eijkemans, chair, biostatistician;
· Dr. P. Blankestijn,, nephrologist;
· Prof. dr. L.J. Bont, pediatrician;
· Dr. M. Langenberg, oncologist;
· Prof. dr. G.J. de Borst, surgeon.

Contact: Dr. G.C.M. van Baal, secretary.

At the moment we are looking for a temporary project specific member as an addition to the DSMB.

This trial will be monitored by the internal DSMB of UMC Utrecht, following their procedures. A report on 1. progress of the study (accrual, quality of the data), and 2. safety will be submitted 1 time each year. Based on this information, the internal DSMB will advise to continue the study without adjustments to the protocol, continue after adjustments, or to stop the study. The advice will only be sent to the sponsor of the study. Should the sponsor decide not to fully implement the advice of the DSMB, the sponsor will send the advice to the

reviewing METC, including a note to substantiate why (part of) the advice of the DSMB will not be followed.

The internal DSMB will monitor the following aspects of the study:
1. Progress: Information on accrual (planned and actual), information on quality of the data (% missing etc.), randomization (check on stratification and baseline variables), losses to follow up with reasons.
2. Safety: Evidence for significant treatment harm: number death, SAEs.
The internal DSMB may also advise on protocol modifications suggested by investigators or sponsors and assess impact and relevance of external evidence.

8. STATISTICAL ANALYSIS

The primary analysis will be based on the intention-to-treat principle (complementary per protocol). If missing values are encountered we will consider using a multiple imputation procedure. Patients without any study activities – from the rTMS treatment – will be replaced.

8.1 Primary study parameter(s)

ARAT scores will be statistically analyzed using repeated measures ANOVA with ‘time’ (different time-points before and after treatment) as within-subject factor and ‘treatment’ (real cTBS vs. sham cTBS) as between-subject factor. Paired t-tests with correction for multiple comparisons will be used for post hoc analysis. Before entering the data in ANOVA, we will check for normal distribution with the Kolmogorov–Smirnov test. Alternatively, Wilcoxon signed-rank tests will be used to analyze ARAT scores.

8.2 Secondary study parameter(s)

Secondary outcome parameters like the additional sensorimotor function tests, and disability/quality of life scores (HADS, BI, SIS) as well as corticospinal excitability and intracortical inhibition measured from diagnostic TMS, will be analyzed in the same way as described for ARAT scores.

MRI data will be processed and analyzed with standard procedures for image registration; statistical mapping of functional activation and connectivity maps; and calculation of tissue diffusion parameters. The volume of functional activation, the degree of functional connectivity and diffusion fractional anisotropy will be quantified in different regions-of-interest of the sensorimotor network. Statistical analysis of imaging parameters will involve repeated measures ANOVA with ‘time’ (different time-points before and after treatment) as within-subject factor and ‘treatment’ (real cTBS vs. sham cTBS) as between-subject factor, followed by post hoc t-testing with correction for multiple comparisons. For predictive modeling we will employ GLM-based algorithms[55], but we may also use alternative algorithms that we have recently tested on their ability to predict infarction based on multiparametric MRI.[56] All necessary software protocols for image processing and analyses are available at our institute.

The open-ended questions provided qualitative data that will be analysed using thematic analysis. Key themes or categories will be identified and coded by reading and rereading the responses from all the interviewed patients.

8.3 Other study parameters

These parameters will be used to check if the groups are comparable on baseline. In case of significant differences between subjects on parameters (which are relevant for the scores on the dependent variable) then a covariance analysis will be performed.

8.4 Interim analysis (if applicable)

Interim analysis will not be performed, because of the negligible risk of the study. The DSMB will only perform ongoing safety surveillance.

9. ETHICAL CONSIDERATIONS

9.1 Regulation statement

This study will be conducted according to the principles of the Declaration of Helsinki (64th WMA General Assembly, Fortaleza, Brazil, October 2013) and in accordance with the Medical Research Involving Human Subjects Act (WMO).

9.2 Recruitment and consent

Eligible ischemic and hemorrhagic stroke patients will be approached for our study in consultation with the involved neurologist or rehabilitation physician. Subsequently, the researchers involved will provide written information as well as a detailed explanation of all the procedures (Patient Information Letter, E1). It is important that the patient have read the letter, have formed an opinion about it and may have discussed this with relatives. After permission by the patient, he/she will be recruited as soon as possible to participate in this RCT. Before actual examination, the researcher will repeat the study information and patients will (again) be informed about the possibility to ask questions to the researcher, and the option to withdrawal from the study at any time will be emphasized. Mental competence is determined by the treating physician beforehand. Participation is only valid after handing in written consent, signed in the presence of the researcher. If the patient is not able to write down the required information (because of motor disabilities), a relative can fill out the informed consent. A copy of the written consent will be given to the patient. The process of obtaining informed consent should be documented in the medical record of the subject. cTBS intervention will only be performed at rehabilitation center De Hoogstraat.

9.3 Objection by minors or incapacitated subjects (if applicable)

Not applicable.

9.4 Benefits and risks assessment, group relatedness

The risk to participants is considered negligible, while the potential benefits are considerable. Sensorimotor function testing and upper limb training are not accompanied by any side effects. Some patients experience a slight painless tap on the head during TMS and a smaller percentage experiences mild and transient headache.[60] During MRI, patients may experience claustrophobic feelings.

We are aware of the burden on patients of subacute intervention and testing in their early stage of recovery. However, evidence is compelling that functional outcome is largely

determined in the first weeks after stroke. Consequently, therapeutic efficacy of interventions is expected to be largest in the early phase after stroke. Next to the opportunity to evaluate early applied recovery-promoting therapy, we expect to obtain new insights into mechanisms contributing to functional recovery after paresis of the upper limb, which is a prerequisite for future optimal treatment planning. This research, however, can only be performed with these patient groups.

9.5 Compensation for injury

The sponsor (also) has an insurance which is in accordance with the legal requirements in the Netherlands (Article 7 WMO). This insurance provides cover for damage to research subjects through injury or death caused by the study.
The insurance applies to the damage that becomes apparent during the study or within 4 years after the end of the study.

1. The insurance applies to the damage that becomes apparent during the study or within 4 years after the end of the study. € 650.000,-- (i.e. six hundred and fifty thousand Euro) for death or injury for each subject who participates in the Research;
2. € 5.000.000,-- (i.e. five million Euro) for death or injury for all subjects who participate in the Research;
3. € 7.500.000,-- (i.e. seven and a half million Euro) for the total damage incurred by the organisation for all damage disclosed by scientific research for the Sponsor as ‘verrichter’ in the meaning of said Act in each year of insurance coverage.

9.6 Incentives (if applicable)

Not applicable.

10. ADMINISTRATIVE ASPECTS, MONITORING AND PUBLICATION

10.1 Handling and storage of data and documents

All personal data will be handled confidentially according to the EU General Data protection regulation (GDPR). Data will be entered into a digital database Open Clinica by the investigators who had direct contact with the subject. The video images of the skilled reaching task are collected and saved on a secured, local server from De Hoogstraat after the video recording. The video images are immediately deleted from the video camera after transfer to the computer. Identifying confidential information and/or health care services will be deleted from the transcript of the interviews.

Each subject will be given an identification code reflecting the group and position of the subject in the database. Only the investigators have excess to the key of the code. Raw data will be stored in one central archive (at a locker in Kenniscentrum De Hoogstraat) and will be safeguarded by the project coordinator. The raw data will be stored for as long as the data is used for research purposes and for 15 years minimally. The digital database will be accessible to all the investigators participating in this research project.

All baseline data, single-pulse TMS output, sensorimotor function tests data and data from questionnaires will be recruited (and stored) at de Hoogstraat. Some informed consents will be obtained at de Hoogstraat as well. The other part of the informed consents and all MRI data will be obtained at the UMC.

10.2 Monitoring and Quality Assurance

This study has a negligible risk, based on the risk classification of the Dutch Federation of University Medical Centers[96] (NFU). Intensity of monitoring is based on the risk classification. Monitoring will be done by an independent and qualified monitor. The monitoring at the UMC/De Hoogstraat will be done by a central internal monitor of the Julius Center'. This person is not involved in the design and execution of the study.

Details can be found in a separate monitoring plan (K6).

10.3 Amendments

Amendments are changes made to the research after a favourable opinion by the accredited METC has been given. All amendments will be notified to the METC that gave a favourable opinion. All substantial amendments will be notified to the METC and to the competent authority.Non-substantial amendments will not be notified to the accredited METC and the competent authority, but will be recorded and filed by the sponsor.

10.4 Annual progress report

The sponsor/investigator will submit a summary of the progress of the trial to the accredited METC once a year. Information will be provided on the date of inclusion of the first subject, numbers of subjects included and numbers of subjects that have completed the trial, serious adverse events/ serious adverse reactions, other problems, and amendments.

10.5 Temporary halt and (prematurely) end of study report

The investigator/sponsor will notify the accredited METC of the end of the study within a period of 8 weeks. The end of the study is defined as the last patient's last visit. The sponsor will notify the METC immediately of a temporary halt of the study, including the reason of such an action. In case the study is ended prematurely, the sponsor will notify the accredited METC within 15 days, including the reasons for the premature termination. Within one year after the end of the study, the investigator/sponsor will submit a final study report with the results of the study, including any publications/abstracts of the study, to the accredited METC.

10.6 Public disclosure and publication policy

Results of the described project will be disclosed and published in peer-reviewed international scientific journals.

11. STRUCTURED RISK ANALYSIS

11.1 Potential issues of concern

a. Level of knowledge about mechanism of action

TMS induces current in the cortex with a coil that generates a magnetic field.[16] Delivery of repetitive trains of TMS (rTMS) at high-frequency enhances cortical excitability, while repetitive low-frequency TMS suppresses cortical excitability.[16] Patterned protocols consisting of short trains of high-frequency TMS (30-100 Hz) in the theta-frequency range (4-7 Hz) (theta-burst stimulation (TBS)[17] have been shown to provide effective and reliable paradigms for excitatory (intermittent TBS (iTBS)) or inhibitory (continuous TBS (cTBS)) brain stimulation, with lasting effects that exceed those induced by standard rTMS protocols.[18] We don't know the underlying mode of action of cTBS – specifically its influence on neural network reorganization over prolonged periods. The modulatory effects of rTMS depend on the chosen stimulation parameters like intensity, frequency, number of sessions, positioning of coil on the head, et cetera. Several studies highlight the role of *y*-aminobutyric acid receptor (GABA-r) modulation, N-methyl-D-aspartate receptors (NMDA-r) and expression of immediate early genes (IEGs) proteins.[17, 67-71]

<u>b. Previous exposure of human beings with the test product(s) and/or products with a similar biological mechanism</u>

The most common non-invasive brain stimulation approaches involve transcranial electrical stimulation, transcranial (direct) current stimulation, transcranial magnetic stimulation and repetitive transcranial magnetic stimulation.[18] The early approach of transcranial electrical stimulation, an uncomfortable method, applied high-voltage electrical stimulation through electrodes on the scalp. Transcranial direct current stimulation (tDCS) is the most common form of transcranial current stimulation. This method induces polarity specific changes delivered via scalp electrodes. tDCS is known for its simplicity and relative low cost, but moderate temporal and focal resolution.[15, 18, 60] Transcranial magnetic stimulation (TMS) uses the principle of a varying magnetic field to induce small electrical currents in the brain on the site of the stimulation. The magnetic pulses can also be applied in a repetitive (pulse) mode, known as rTMS. In contrast to tDCS, rTMS has good temporal and spatial resolution, but is has a relatively complex and expensive setup.[15-17, 51] TMS is most commonly used to study brain plasticity and physiology and rTMS to evoke neuroplasticity and neuromodulation. Nowadays different theta burst protocols are being used, a repetitive application of burst-trains. Theta burst stimulation is characterized by its short duration and longer lasting aftereffects.[15-18, 51, 60]

<u>c. Can the primary or secondary mechanism be induced in animals and/or in *ex-vivo* human cell material?</u>

rTMS can be given to animals, and the effects can be found on neural network reorganization and behaviour. The primary or secondary mechanism cannot be induced in ex-vivo human cell material. The short duration time of TBS protocols makes it possible to stimulate non-anesthetized animals.[72] A limitation for rTMS study in small laboratory animals, like rodents, is the fact that focal stimulation of distinct rodent brain areas is not possible, due to limitations in coil size.[72, 102] The size and thickness of the brain determines the induced current density distribution and the spatial selectivity of the impact.[51] Advanced sensorimotor function testing and measuring disability and quality of life is not possible in animals.

<u>d. Selectivity of the mechanism to target tissue in animals and/or human beings</u>

Motor cortical stimulation has been the main focus of most previous studies. The primary motor cortex, ipsilesional as well as contralesional, has been used as target. Inhibitory stimulation of the contralesional hemisphere can be used to reduce the undesirable inhibitory drive to the affected hemisphere, whereas excitatory stimulation of the ipsilesional hemisphere can be used to enhance the reduced participation of the affected hemisphere. The used mode, intermittent or continuous for TBS, determines the induced changes in the cortex (excitatory or inhibitory).[12, 15]

<u>e. Analysis of potential effect</u>

The literature shows that non-invasive brain stimulation (NIBS) techniques have therapeutic effects in a wide range of patient populations, such as depression, aphasia, migraine, motor dysfunction and epilepsy.[87-93] When the safety guidelines are followed, these techniques are generally safe.[51, 94] The safety guidelines are a comprehensive list of ethical issues, stimulation parameters, physiological monitoring, screening questionnaire for patients et cetera. The most serious TMS-related side-effect, the occurrence of seizures, has been extremely rare.[51] The crude risk of seizure is approximately 0.02% per session of TBS and 1.1% for mild adverse events.[58] Examples of adverse events during or immediately after TBS in 67 protocols (4%) are mild headache, nonspecific discomfort (tinnitus patients), mild discomfort (neck pain and lightheadedness).[58]

<u>f. Pharmacokinetic considerations</u>

Not applicable.

g. Study population

The research subjects are adult patients with a first-ever unilateral ischemic and hemorrhagic stroke with mild to moderate mono- or hemiparesis of the arm, within the first 1-2 weeks after stroke onset. Chronic patients will show a more stable impairment, but in the subacute stage of recovery much of the neural reorganization is expected to occur.[6, 15]

h. Interaction with other products

Not applicable.

i. Predictability of effect

In the time period of the introduction of TBS until now, TBS techniques have proved to be a powerful therapeutic tool. TBS has an advantage over other non-invasive brain stimulation protocols, especially in clinical practice, due to its short duration time and low intensity stimulus pulses.[72] Several studies have demonstrated that reducing the excitability of the contralesional cortex with cTBS can improve motor outcome.[62] Furthermore, the first month post stroke is the ideal time window for neurorehabilitation.

j. Can effects be managed?

The exclusion criteria are described as such that patients with counterindications for TMS are not included. In addition, there are also multiple safety guidelines and questionnaires (for the use in clinical practice) available to fill in prior to the stimulation. The side-effects of rTMS are well described and very low. The TMS stimulation takes places in rehabilitation center De Hoogstraat, where the patients are hospitalized. If there is a rare case reporting side-effects, the treating physician will be informed and asked to treat this patient.

11.2 Synthesis

Sensorimotor function testing and upper limb training are not accompanied by any side effects. During MRI, patients may experience claustrophobic feelings.

The occurrence of seizures, the most serious acute TMS-related side effect, has been reported, but especially before the implementation of safety guidelines. Safety and ethical guidelines have been made during consensus conferences for TMS stimulation in therapeutics and academic goals. [51, 85] The risk of TMS-induced seizures is very low, with regard to the large amount of participants and patients who have undergone rTMS stimulation. Seizures are only reported during or immediately after trains of rTMS, not

during the aftereffects.[51, 95] TBS induced seizure has only occurred once in more than 4500 sessions, resulting in a crude risk of approximately 0.02%. In other high frequency rTMS protocols the reported seizure rate is less than 0.1%.[58]

The most common reported adverse events in TBS are almost the same as the ones reported in rTMS, namely transient headache and neck pain. Up to 40% of the patients undergoing rTMS reported these adverse events, in contrast to (less than) 3% of the patients receiving TBS.[51, 58]

Concluding, the risks of side effects during and immediately after TBS are negligible. The reported headache and neck pain were temporary and not harmful. This is doable given the potential informative gains from this study. Furthermore, there are multiple safety guidelines available, a consensus-based screening questionnaire (prior to stimulation), strict in- and exclusion criteria for a complete overview.[58, 85]

12. REFERENCES

1. Kwakkel G, Kollen BJ, Wagenaar RC. Long term effects of intensity of upper and lower limb training after stroke: A randomised trial. J Neurol Neurosurg Psychiatry. 2002;72:473-479

2. Mayo NE, Wood-Dauphinee S, Ahmed S, Gordon C, Higgins J, McEwen S, Salbach N. Disablement following stroke. Disabil Rehabil. 1999;21:258-268

3. Wardlaw JM, Murray V, Berge E, Del Zoppo GJ. Thrombolysis for acute ischaemic stroke. Cochrane Database Syst Rev. 2009:CD000213

4. Fonarow GC, Smith EE, Saver JL, Reeves MJ, Bhatt DL, Grau-Sepulveda MV, Olson DM, Hernandez AF, Peterson ED, Schwamm LH. Timeliness of tissue-type plasminogen activator therapy in acute ischemic stroke: Patient characteristics, hospital factors, and outcomes associated with door-to-needle times within 60 minutes. Circulation. 2011;123:750-758

5. Cramer SC, Sur M, Dobkin BH, O'Brien C, Sanger TD, Trojanowski JQ, Rumsey JM, Hicks R, Cameron J, Chen D, Chen WG, Cohen LG, deCharms C, Duffy CJ, Eden GF, Fetz EE, Filart R, Freund M, Grant SJ, Haber S, Kalivas PW, Kolb B, Kramer AF, Lynch M, Mayberg HS, McQuillen PS, Nitkin R, Pascual-Leone A, Reuter-Lorenz P, Schiff N, Sharma A, Shekim L, Stryker M, Sullivan EV, Vinogradov S. Harnessing neuroplasticity for clinical applications. Brain. 2011;134:1591-1609

6. Dancause N, Nudo RJ. Shaping plasticity to enhance recovery after injury. Prog Brain Res. 2011;192:273-295

7. Dimyan MA, Cohen LG. Neuroplasticity in the context of motor rehabilitation after stroke. Nat Rev Neurol. 2011;7:76-85

8. Iadecola C, Anrather J. Stroke research at a crossroad: Asking the brain for directions. Nat Neurosci. 2011;14:1363-1368

9. Krakauer JW, Carmichael ST, Corbett D, Wittenberg GF. Getting neurorehabilitation right: What can be learned from animal models? Neurorehabil Neural Repair. 2012

10. Hoyer EH, Celnik, PA. Understanding and enhancing motor recovery after stroke using transcranial magnetic stimulation. Restor Neurol Neurosci 2011;29:395-409

11. Le Q, Qu Y, Tao Y, Zhu S: Effects of repetitive transcranial magnetic stimulation on hand function recovery and excitability of the motor cortex after stroke: A meta-analysis. Am J Phys Med Rehabil 2014;93:422-430

12. Hummel FC, Cohen LG: Noninvasive brain stimulation: a new strategy to improve neurorehabilitation after stroke? Lancet Neurol 2006;5:708-712

13. Liew S, Santarnecchi E, Buch ER, Cohen LG. Non-invasive brain stimulation in neurorehabilitation: local and distant effects on motor recovery. Frontiers in Neuroscience 2014;8:1-15

14. Simonetta-Moreau M. Non-invasive brain stimulation (NIBS) and motor recovery after stroke. Annals of Physical and Rehabilitation Medicine 2014;57:530-542
15. Wessel MJ, Zimerman M, Hummel FC. Non-invasive brain stimulation: An interventional tool for enhancing behavioral training after stroke. Front Hum Neurosci 2015;9:265
16. Hallett M. Transcranial magnetic stimulation: A primer. Neuron. 2007;55:187-199
17. Huang Y, Edwards MJ, Rounis E, Bhatia, KP, Rothwell JC. Theta burst stimulation of the human motor cortex. Neuron 2005;45:201-206
18. Rossini, PM, Burke D, Chen R, Cohen LG, Daskalakis Z, Di Iorio R et al. Non-invasive electrical and magnetic stimulation of the brain, spinal cord, roots and peripheral nerves: Basic principles and procedures for routine clinical and research application. An updated report from an I.F.C.N. Committee. Clin Neurophysiol 2015;126:1071-1107
19. Delvaux V, Alagona G, Gérard P, De Pasqua V, Pennisi G, de Noordhout AM. Clin Neuropsychiol 2003;114:1217-1225
20. Ward, NS, Brown, MM, Thompson, AJ, Frackowiak, RS. Neural correlates of motor recovery after stroke: a longitudinal fMRI study. Annals of Physical and Rehabilitation Medicine 2003;126:2476-2496
21. Murase, N, Duque, J, Mazzocchio, R, Cohen, LG. Influence of interhemispheric interactions on motor function in chronic stroke. Ann Neurol 2004;55:400-409
22. Duque, J, Hummel, F, Celnik, P, Murase, N, Mazzocchio, R, Cohen, LG. Transcallosal inhibition in chronic subcortical stroke. Neuroimage 2005;28:940-946
23. Bloom, JS, Hynd, GW. The role of the corpus callosum in interhemispheric transfer of information: excitation or inhibition? Neuropsychol Rev 2005;15:59-71
24. Nowak, DA, Grefkes, C, Ameli, M, Fink, GR. Interhemispheric competition after stroke: brain stimulation to enhance recovery of function of the affected hand. Neurorehabil Neural Repair 2009;23:641-656
25. Grefkes, C, Nowak, DA, Eickhoff, SB, Dafotakis, M, Kust, J, Karbe, H, et al. Cortical connectivity after subcortical stroke assessed with functional magnetic resonance imaging. Ann Neurol 2008;63:236-246
26. Chang WH, Kim YH, Bang OY, Kim ST, Park YH, Lee PK. Long-term effects of rtms on motor recovery in patients after subacute stroke. J Rehabil Med. 2010;42:758- 764
27. Khedr EM, Etraby AE, Hemeda M, Nasef AM, Razek AA. Long-term effect of repetitive transcranial magnetic stimulation on motor function recovery after acute ischemic stroke. Acta Neurol Scand. 2010;121:30-37
28. Hoyer EH, Celnik PA. Understanding and enhancing motor recovery after stroke using transcranial magnetic stimulation. Restor Neurol Neurosci. 2011;29:395-409
29. Chang WH, Kim YH, Bang OY, Kim ST, Park YH, Lee PK. Long-term effects of rtms on motor recovery in patients after subacute stroke. J Rehabil Med. 2010;42:758- 764

30. Khedr EM, Etraby AE, Hemeda M, Nasef AM, Razek AA. Long-term effect of repetitive transcranial magnetic stimulation on motor function recovery after acute ischemic stroke. Acta Neurol Scand. 2010;121:30-37
31. Ziemann, U, Paulus, W, Nitsche, MA, Pascual-Leone, A, Byblow, WD, Berardelli, A, et al. Consensus: motor cortex plasticity protocols. Brain stimulat. 2008;1:164-182
32. Baumer, T, Lange, R, Liepert, J, Weiller, C, Siebner, HR, Rothwell, JC, et al. Repeated premotor rTMS leads to cumulative plastic changes of motor cortex excitability in humans. Neuroimage 2003; 20:550-560
33. Fritsch, B, Reis, J, Martinowich, K, Schambra, HM, Ji, Y, Cohen, LG, et al. Direct current stimulation promotes BDNF-dependent synaptic plasticity: potential implications for motor learning. Neuron 2010;66:198-204
34. Ackerley, SJ, Stinear, CM, Barber, PA, Byblow, WD. Priming sensorimotor cortex to enhance task-specific training after subcortical stroke. Clincal Neurophysiology 2014;125:1451-1458
35. Talelli, P, et al. Theta burst stimulation in the rehabilitation of the upper limb: A semirandomized, placebo-controlled trial in chronic stroke patients. Neurorehabilitation and Neural Repair 2012;26:976-987
36. Meehan, SK, Dao, E, Meghan, A, Linsdell, MA, Boyd, LA. Continuous theta burst stimulation over the contralesional sensory and motor cortex enhances motor learning post-stroke. Neurosci Lett 2011;500:26-30
37. Di Lazzaro, V, et al. Inhibitory theta burst stimulation of affected hemisphere in chronic stroke: A proof of principle, sham-controlled study. Neurosci Lett 2013;553:148-152
38. Huynh, W, Vucic, S, Krishnan, AV, Lin, CS, Kiernan, MC. Exploring the evolution of cortical excitability following acute stroke. Neurorehabilitation and Neural Repair 2015;1-14
39. Amengual, JL, et al. Prognostic value of cortically induced motor evoked activity by TMS in chronic stroke: caveats from a revealing single clinical case. BMC Neurol. 2012;12:35
40. Yamada, N, et al. Continuous theta-burst stimulation combined with occupational therapy for upper limb hemiparesis after stroke: A preliminary study. Act Neurol Belg 2014;114:279-284
41. Silasi, G, Murphy, TH. Stroke and the connectome: How connectivity guides therapeutic intervention. Neuron 2014;83:1354-1368
42. Dijkhuizen, RM, Zaharchuk, G, Otte, WM. Assessment and modulation of resting-state neural networks after stroke. Curr Opin Neurol;27:637-643
43. Calautti C, Baron JC. Functional neuroimaging studies of motor recovery after stroke in adults: A review. Stroke. 2003;34:1553-1566
44. Dijkhuizen RM, Singhal AB, Mandeville JB, Wu O, Halpern EF, Finklestein SP, Rosen BR, Lo EH. Correlation between brain reorganization, ischemic damage, and neurologic

status after transient focal cerebral ischemia in rats: A functional magnetic resonance imaging study. J Neurosci. 2003;23:510-517
45. Carter AR, Astafiev SV, Lang CE, Connor LT, Rengachary J, Strube MJ, Pope DL, Shulman GL, Corbetta M. Resting interhemispheric functional magnetic resonance imaging connectivity predicts performance after stroke. Ann Neurol. 2010;67:365-375
46. van Meer MP, van der Marel K, Wang K, Otte WM, El Bouazati S, Roeling TA, Viergever MA, Berkelbach van der Sprenkel JW, Dijkhuizen RM. Recovery of sensorimotor function after experimental stroke correlates with restoration of resting-state interhemispheric functional connectivity. J Neurosci. 2010;30:3964-3972
47. Van Meer MP, Otte WM, Van der Marel K, Nijboer CH, Kavelaars A, Berkelbach van der Sprenkel JW, Viergever MA, Dijkhuizen RM. Extent of bilateral neuronal network reorganization and functional recovery in relation to stroke severity. J Neurosci. 2012;32:4495-4507
48. Dancause N, Nudo RJ. Shaping plasticity to enhance recovery after injury. Prog Brain Res. 2011;192:273-295
49. Goldsworthy, MR, Müller-Dahlhaus, F, Ridding, MC, Ziemann, U. Inter-subject variability of LTD-like plasticity in human motor cortex: A matter of preceding motor activation. Brain Stim 2014;7:864-870
50. De Weijer AD, Bloemendaal M, Bakker EJ, Klomp DW, Sommer IE, Bestmann S, Neggers SF. Resting-state tms-fmri to fef, sef and m1 using flex coils and parallel imaging. Neuroscience 2011, 41st Annual Meeting of the Society for Neuroscience. 2011:619.609
51. Rossi, S, Hallett, M, Rossini, PM, Pascual-Leone, A. Safety, ethical considerations, and application guidelines for the use of transcranial magnetic stimulation in clinical practice and research. Clin Neurophysiol 2009;120:2008-2039
52. Lefaucheur, J, et al. Evidence-based guidelines on the therapeutic use of repetitive transcranial magnetic stimulation (rTMS). Clin Neurophysiol 2014;125:2150-2206
53. Kwakkel G, Meskers CG, van Wegen EE, Lankhorst GJ, Geurts AC, van Kuijk AA, Lindeman E, Visser-Meily JMA, de Vlugt E, Arendzen JH. Impact of early applied upper limb stimulation: The explicit-stroke programme design. BMC Neurol. 2008;8:49
54. van der Lee JH, Roorda LD, Beckerman H, Lankhorst GJ, Bouter LM. Improving the action research arm test: A unidimensional hierarchical scale. Clin Rehabil. 2002;16:646-653
55. Wu O, Dijkhuizen RM, Sorensen AG. Multiparametric magnetic resonance imaging of brain disorders. Top Magn Reson Imaging. 2010;21:129-138
56. Bouts MJ, Tiebosch IA, Zwartbol R, Hoogveld E, Wu O, Dijkhuizen RM. Early prediction of salvageable tissue with multiparametric mri-based algorithms after experimental ischemic stroke. Proc Intl Soc Mag Reson Med. 2011;19:2141

57. Wardlaw, JM, Murray, V, Berge, E, Del Zoppo, GJ. Thrombolysis for acute ischaemic stroke. Cochrane Database of Systematic Reviews 2016;7:CD000213
58. Oberman, L, Edwards, D, Eldaief, M, Pascual-Leone, A. Safety of theta burst transcranial magnetic stimulation: A systematic review of the literature. J Clin Neurophysiol 2011;28:67-74
59. Hong, YH, Wu, SW, Pedapati, EV, Horn, PS, Huddleston, DA, Laue, CS, Gilbert, DL. Safety and tolerability of theta burst stimulation vs. single and paired pulse transcranial magnetic stimulation: a comparative study of 165 pediatric subjects. Front Hum Neurosci 2015;9:29
60. Krishnan, C, Santos, L, Peterson, MD, Ehinger, M. Safety of noninvasive brain stimulation in children and adolescents. Brain Stimulation 2015;8:76-87
61. Maizey, L, Allen, CPG, Dervinis, M, Verbruggen, F, Varnava, A, Kozlov, M, Adams, RC, Stokes, M, Klemen, J, Bungert, A, Hounsell, CA, Chambers, CD. Comparative incidence rates of mild adverse effects to transcranial magnetic stimulation. J Clin Neurophysiol 2013;124:536-544
62. Hsu, WY, Cheng, CH, Liao, KK, Lee, IH, Lin, YY. Effects of repetitive transcranial magnetic stimulation on motor functions in patients with stroke: A meta-analysis. Stroke 2012;43:1849-1857
63. Mathiowetz, M, Weber, K, Kashman, N, Volland, G. Adult norms for Nine Hole Peg Test of finger dexterity. Occup Ther J Res. 1985;5:24-38
64. Badke, MB, Duncan, PW. Patterns of rapid motor responses during postural adjustments when standing in healthy subjects and hemiplegic subjects. Phys Ther 1983;63:13-20
65. Duncan, PW, Propst, M, Nelson, SG. Reliability of the Fugl-Meyer assessment of sensorimotor recovery following cerebrovascular accident. Phys Ther 1983;63:1606-1610
66. KNGF-richtlijn Beroerte: verantwoording en toelichting, map K. p. 71-72 https://www.kngfrichtlijnen.nl/index.php?NODE=2004&richtlijn=5&versie=12&hoofdstuk=214
67. Cárdenas-Morales, L, Nowak, DA, Kammer, T, Wolf, RC, Schönfeldt-Lecuona, C. Mechanisms and applications of theta-burst rTMS on the human motor cortex. Brain Topogr 2010;22:294-306
68. Rossini, PM, Rossi, S. Transcranial magnetic stimulation – diagnostic, therapeutic and research potential. Neurology 2007;68:484-488
69. Di Lazzaro, V, Pilato, F, Dileone, M, Profice, P, Oliviero, A, Mazzone, P, Insola, A, Ranieri, F, Meglio, M, Tonali, PA, Rothwell, JC. The physiological basis of the effects of intermittent theta burst stimulation of the human motor cortex. J Physiol 2008;586:3871-3879
70. Thickbroom, GW. Transcranial magnetic stimulation and synaptic plasticity: experimental framework and human models. Exp Brain Res 2007;180:583-593
71. Aydin-Abdin, S, Trippe, J, Funke, K, Eysel, UT, Benali, A. High and low-frequency

repetitive transcranial magnetic stimulation differentially activates c-Fos and zif268 protein expression in the rat brain. Exp Brain Res 2008;188:249-261
72. Suppa, A, Huang, YZ, Funke, K, Ridding, MC, Cheeran, B, Di Lazzaro, V, Ziemann, U, Rothwell, JC. Ten years of theta burst stimulation in humans: Established knowledge, unknowns and prospects. Brain Stimulation 2016; doi: 10.1016/j.brs.2016.01.006
73. Kasner, SE. Clinical interpretation and use of stroke scales. Lancet Neurology 2006;5:603-612
74. Bovend'Eerdt, TJ, Dawes, H, Johansen-Berg, H, Wade, DT. Evaluation of the modified Jebsen test of hand function and the University of Maryland arm questionnaire for stroke. Clinical Rehabilitation 2004;18:195-202
75. Mahoney FI, Barthel DW. Functional evaluation. The Barthel Index. MD State Med J 1965;14 61-65
76. Granger CV, Dewis LS, Peters NC, Sherwood CC, Barrett JE. Stroke rehabilitation: analysis of repeated Barthel index measures. Arch Phys Med Rehabil 1979;60:14-17
77. Sulter G, Steen C, De KJ. Use of the Barthel index and modified Rankin scale in acute stroke trials. Stroke 1999;30:1538-1541
78. Duncan, PW, Bode, RK, et al. Rasch analysis of a new stroke-specific outcome scale: the Stroke Impact Scale. Arch Phys Med Rehabil 2003;84:950-963
79. Zigmond, AS, Snaith, RP. The Hospital Anxiety and Depression Scale. Acta psychiatrica scandinavica 1983;67:361-370
80. Maathuis, R, Storm, M, Visser-de Heus, B, Visser-Meily, JMA, Zinger, D. Exercising after a stroke: do it yourself! 1st ed. Utrecht: Kenniscentrum De Hoogstraat; 2013
81. Kwakkel, G, van Peppen, R, Wagenaar, RC, Wood Dauphinee, S, Richards, C, Ashburn, A. Effects of augmented exercise therapy time after stroke: a meta-analysis. Stroke 2004;35:2529-2539
82. Kwakkel, G. Impact of intensity of practice after stroke: issues for consideration. Disabil Rehabil 2006;28:823-830
83. Bhogal, S, Teasell, R, Speechley, M, Albert, M. Intensity of aphasia therapy, imoact on recovery. Stroke 2003;34:987-993
84. van de Port, GL, Valkenet, K, Schuurmans, M, Visser-Meily, JMA. How to increase activity level in the acute phase after stroke. J Clin Nurs 2012;21:3574-3578
85. Rossi, S, Hallett, M, Rossini, PM, Pascual-Leone, A. Screening questionnaire before TMS: an update. Clin Neurophysiology 2011;122:1686
86. Zimmermann-Schlatter, A, Schuster, CA, Puhan, M, Siekierska, E, Steurer, J. Efficacy of motor imagery in post-stroke rehabilitation: a systematic review. Journal of NeuroEngineering and Rehabilitation 2008;5:8
87. Cotelli, M, Manenti, R, Petesi, M, et al. Treatment of primary progressive aphasias by

transcranial direct current stimulation combined with language training. J Alzheimers Dis 2014;39:799-808

88. Berthier, ML, Pulvermuller, F. Neuroscience insights improve neurorehabilitation of poststroke aphasia. Nat Rev Neurol 2011;7:86-97

89. Berlim, MT, Van den Eynde, F, Daskalakis, ZJ. Clinical utility of transcranial direct current stimulation (tDCS) for treating major depression: a systematic review and meta-analysis of randomized, double-blind and sham-controlled trials. J Psychiatr Res 2013;47:1-7

90. Wall, CA, Croarkin, PE, McClintock, SM, et al. Neurocognitive effects of repetitive transcranial magnetic stimulation in adolescents with major depressive disorder. Front Psychiatry 2013;4:165

91. Varga, ET, Terney, D, Atkins, MD, et al. Transcranial direct current stimulation in refractory continuous spikes and waves during slow sleep: a controlled study. Epilepsy Res 2011;97:142-150

92. Wu, AD, Fregni, F, Simon, DK, Deblieck, C, Pascual-Leone, A. Noninvasive brain stimulation for Parkinson's disease and dystonia. Neurotherapeutics 2008;5:345-361

93. Lipton, RB, Dodick, DW, Silberstein, SD, et al. Single-pulse transcranial magnetic stimulation for acute treatment of migraine with aura: a randomized double-blind, parallel-group, sham-controlled trial. Lancet Neurol 2010;9:373-380

94. Poreisz, C, Boros, K, Antal, A, Paulus, W. Safety aspects of transcranial direct current stimulation concerning healthy subjects and patients. Brain Res Bull 2007;72:208-214

95. Wassermann, EM, Grafman, J, Berry, C, Hollnagel, C, Wild, K, Clark, K, et al. Use and safety of a new repetitive transcranial magnetic stimulator. Electroencephalogr Clin Neurophysiol 1996;101:412–417

96. Nederlandse Federatie van Universitair Medische Centra. Kwaliteitsborging mensgebonden onderzoek 2.0. Bouma, C, Piekhaar, M, editors. Nederlandse Federatie van Universitair Medische Centra; 2012

97. Kwakkel, G, Kollen, BJ, van der Gron, J, Prevo, AJH. Probability of regaining dexterity in the flaccid upper limb: impact of severity of paresis and time since onset in acute stroke. Stroke 2003;34:2181-2186

98. Faul, F, Erdfelder, E, Lang, AG, Buchner, A. G*Power 3: A flexible statistical power analysis program for the social, behavioral, and biomedical sciences. Behavior Research Methods 2007;39:175-191

99. Ermers, W. e.a. Stroke Upper Limb Capacity Scale. Een meetinstrument voor basale tot complexe arm- en handvaardigheid na CVA. EM 2013;41:16-19

100. Houwink, A, Roorda, LD, Smits, W, Molenaar, IW, Geurts, AC. Measuring upper limb capacity in patients after stroke: Reliability and validity of the Stroke Upper Limb Capacity Scale. Arch Phys Med Rehabil. 2011;92:1418-1422

101. Winstein, CJ, Miller, JP, Blanton, S, Taub, E, Uswatte, G, Morris, D, Nichols, D, Wolf, S. Methods for a multisized randomized trial to investigate the effect of constraint-induced movement therapy in improving upper extremity function among adults recovering from a cerebrovascular stroke. The American Society of Neurorehabilitation 2003;17:137-152

102. Wassermann, EM, Lisanby, SH. Therapeutic application of repetitive transcranial magnetic stimulation: a review. Clinical Neurophysiology 2011;112:1367-1377.

103. Buma, FE, Raemaekers, M, Kwakkel, G, Ramsey, NF. Brain function and upper limb outcome in stroke: a cross-sectional fMRI study. PLoS ONE 2015;10:1-18

104. Golicki D, Niewada M, Buczek J. Validity of EQ-5D-5L un stroke. Quality of Life Research 2015:24;845-850

105. Ruff, R, Parker, S. Gender- and age-specific changes in motor speed and eye-hand coordination in adults: normative values for the Finger Tapping and Grooved Pegboard Tests. Perceptual and Motor Skills 1993:76;319-330

B-STARS statistical analysis plan

**Dutch trial register**	NL5952 (NTR6133)
**Funding party**	Netherlands Organization for Scientific Research
**Sponsor**	University Medical Center Utrecht
**Version**	3.0
**Date**	7-5-2021
**Principal Investigator**	Professor J.M.A. Visser-Meily
**Trial statistician**	Dr. R.P.A van Eijk and Dr. W.M. Otte

1. Aim of the study

Despite therapies in the acute phase of stroke, many patients are left with long-term impairment of upper limb function. The B-STARS trial is a randomized sham-controlled clinical trial that investigates whether contralesional inhibitory TMS treatment started in the first three weeks after stroke onset improves upper limb function in patients with arm weakness.

2. Study design

The B-STARS study is a randomized, sham-controlled clinical trial with a single-blind intervention and a double-blind primary outcome evaluation. Patients were randomly assigned to ten daily sessions of a variant of inhibitory Transcranial Magnetic Stimulation (TMS), i.e. continuous Theta Burst Stimulation (cTBS), or sham cTBS, of the contralesional primary motor cortex, in addition to standard rehabilitation therapy. Patients were stratified in a high performance group if they were able to voluntarily extend one or more fingers, or in a low performance group if they could not (Kwakkel et al. 2016). The study protocol has been published (van Lieshout et al. 2017).

The primary outcome is the change in the action research arm test (ARAT) score between the baseline measurement and 3 months post-stroke, based on recommendations by the Stroke Recovery and Rehabilitation Roundtable (SRRR)(Kwakkel et al. 2017) and the international classification of functioning, disability and health (ICF) framework(Stucki et al. 2002). The primary outcome measure is assessed by a non-treating rater, blinded to treatment allocation.

A sample of 56 patients was required to reliably determine a treatment effect with an effect size of 0.55(Hsu et al. 2012) with statistical power of 0.8 and alpha of 0.05. A sample of 60 patients, with 30 patients per group, was used in order to account for loss to follow-up. The minimal clinically important difference (MCID) was set at 6 points on the ARAT, in correspondence with previous clinical studies(Kwakkel et al. 2016).

3. Analysis population

The primary analysis will be performed on the intention-to-treat (ITT) population.

A secondary sensitivity analysis will be performed on the per-protocol population, which will exclude patients with protocol violations, and patients in whom the primary endpoint could not be reliably assessed.

4. Data and statistical analysis

4.1. Handling of missing data

Missing data in the ITT population will be imputed using multiple imputations. Ten rounds of imputations will be performed on the complete dataset including additional time points:

immediately post treatment (T1); 1 week after treatment (T2) and 1 month after treatment (T3). ARAT scores will be imputed and the ARAT scores of patients in the sham group, patient, sex, age, stroke severity, stroke type and session will be used as predictors in the imputation model.
Missing data in the per-protocol population will not be imputed.

4.2. Quality control

Prior to unblinding, all data will be checked and potential errors will be corrected if needed. In addition, full analyses will be run based on dummy randomization codes.

5. Planned analyses

Statistical analyses will be performed in SPSS 26.

5.1. Retention

A flow diagram will be constructed, reporting the number of patients who were randomized and treated, and who completed follow-up by treatment group.

5.2. Baseline data

Baseline characteristics will be reported per treatment group without statistical testing between groups. Baseline characteristics will be reported according to available data and recommendations of the SRRR, which include: Sex, age, race, education, risk factors, acute intervention, lesion type, lesion location, impaired side, stroke severity and baseline motor function (Fugl-Meyer (FM)), activity (ARAT, Stroke Upper Limb Capacity Scale (SULCS), Barthel Index (BI), Jebsen Taylor Test (JTT) and Nine Hole Peg Test (NHPT)), as well as participation (Modified Rankin Scale (MRS), Stroke Impact Scale (SIS), EuroQol-5Dimensions added cognition (EQ-5D+). In addition, we provide baseline characteristics on the timing of the TMS intervention, the hospital anxiety and depression scale (HADS), motor-evoked potential presence and the resting motor threshold.

5.3. Analysis of primary outcome

The primary outcome is the change in ARAT score between baseline and 3 month post-stroke. The primary outcome will be analyzed between the two treatment groups, using an analysis of covariance (ANCOVA) with the change in ARAT score between baseline and 3 months post-stroke as the dependent variable, and treatment group (0 = sham, 1 = placebo), baseline ARAT score and stratification (0 = low performance, 1 = high performance) as covariates. The main hypothesis will be tested 2-tailed with an alpha of 0.05. Normality of the residuals will be checked. In case of non-normality, Poisson regression will be used.

A sensitivity analysis will be performed on the per-protocol population with the same statistical methodology.

5.4. Analysis of secondary outcomes

The secondary outcomes are the change between baseline and 3 months post-stroke in each of the two treatment groups on the following scores: FM, SULCS, BI, JTT, NHPT, MRS, SIS and the EQ-5D+. These scores will be evaluated an analysis of covariance (ANCOVA) with the score at 3 months post-stroke as the dependent variable, treatment group as fixed factor and baseline score as covariate. Normality of the residuals will be checked. In case of non-

normality, Poisson regression will be used. All hypothesis will be tested 2-tailed with an alpha of 0.05.

5.5. Adverse events

Occurrences of (serious) adverse events will be reported per treatment group and compared between groups with Fisher's exact tests. Hypotheses will be tested 2-tailed with an alpha of 0.05.

6. References

Hsu W-Y, Cheng C-H, Liao K-K, et al (2012) Effects of repetitive transcranial magnetic stimulation on motor functions in patients with stroke: a meta-analysis. Stroke 43:1849–1857

Kwakkel G, Lannin NA, Borschmann K, et al (2017) Standardized Measurement of Sensorimotor Recovery in Stroke Trials: Consensus-Based Core Recommendations from the Stroke Recovery and Rehabilitation Roundtable. Neurorehabil Neural Repair 31:784–792. https://doi.org/10.1177/1545968317732662

Kwakkel G, Winters C, Van Wegen EEH, et al (2016) Effects of Unilateral Upper Limb Training in Two Distinct Prognostic Groups Early after Stroke: The EXPLICIT-Stroke Randomized Clinical Trial. Neurorehabil Neural Repair 30:804–816. https://doi.org/10.1177/1545968315624784

Stucki G, Cieza A, Ewert T, et al (2002) Application of the International Classification of Functioning, Disability and Health (ICF) in clinical practice. Disabil Rehabil 24:281–282

van Lieshout ECC, Visser-Meily JMA, Neggers SFW, et al (2017) Brain stimulation for arm recovery after stroke (B-STARS): protocol for a randomised controlled trial in subacute stroke patients. BMJ Open 7:e016566. https://doi.org/10.1136/bmjopen-2017-016566

CONSORT 2010 checklist of information to include when reporting a randomised trial*

Section/Topic	Item No	Checklist item	Reported on page No
**Title and abstract**			
	1a	Identification as a randomised trial in the title	1
	1b	Structured summary of trial design, methods, results, and conclusions (for specific guidance see CONSORT for abstracts)	3
**Introduction**			
Background and objectives	2a	Scientific background and explanation of rationale	4
	2b	Specific objectives or hypotheses	4
**Methods**			
Trial design	3a	Description of trial design (such as parallel, factorial) including allocation ratio	5
	3b	Important changes to methods after trial commencement (such as eligibility criteria), with reasons	Protocol
Participants	4a	Eligibility criteria for participants	5
	4b	Settings and locations where the data were collected	5
Interventions	5	The interventions for each group with sufficient details to allow replication, including how and when they were actually administered	5/6
Outcomes	6a	Completely defined pre-specified primary and secondary outcome measures, including how and when they were assessed	6
	6b	Any changes to trial outcomes after the trial commenced, with reasons	NA
Sample size	7a	How sample size was determined	6
	7b	When applicable, explanation of any interim analyses and stopping guidelines	NA
Randomisation:			
Sequence generation	8a	Method used to generate the random allocation sequence	5
	8b	Type of randomisation; details of any restriction (such as blocking and block size)	5
Allocation concealment mechanism	9	Mechanism used to implement the random allocation sequence (such as sequentially numbered containers), describing any steps taken to conceal the sequence until interventions were assigned	5
Implementation	10	Who generated the random allocation sequence, who enrolled participants, and who assigned participants to interventions	5
Blinding	11a	If done, who was blinded after assignment to interventions (for example, participants, care providers, those	5

		assessing outcomes) and how	
	11b	If relevant, description of the similarity of interventions	5/6
Statistical methods	12a	Statistical methods used to compare groups for primary and secondary outcomes	6/7
	12b	Methods for additional analyses, such as subgroup analyses and adjusted analyses	6/7
**Results**			
Participant flow (a diagram is strongly recommended)	13a	For each group, the numbers of participants who were randomly assigned, received intended treatment, and were analysed for the primary outcome	7
	13b	For each group, losses and exclusions after randomisation, together with reasons	7
Recruitment	14a	Dates defining the periods of recruitment and follow-up	7
	14b	Why the trial ended or was stopped	7
Baseline data	15	A table showing baseline demographic and clinical characteristics for each group	8/9
Numbers analysed	16	For each group, number of participants (denominator) included in each analysis and whether the analysis was by original assigned groups	7
Outcomes and estimation	17a	For each primary and secondary outcome, results for each group, and the estimated effect size and its precision (such as 95% confidence interval)	7/8
	17b	For binary outcomes, presentation of both absolute and relative effect sizes is recommended	NA
Ancillary analyses	18	Results of any other analyses performed, including subgroup analyses and adjusted analyses, distinguishing pre-specified from exploratory	7/8
Harms	19	All important harms or unintended effects in each group (for specific guidance see CONSORT for harms)	8
**Discussion**			
Limitations	20	Trial limitations, addressing sources of potential bias, imprecision, and, if relevant, multiplicity of analyses	14
Generalisability	21	Generalisability (external validity, applicability) of the trial findings	13/14
Interpretation	22	Interpretation consistent with results, balancing benefits and harms, and considering other relevant evidence	13/14
**Other information**			
Registration	23	Registration number and name of trial registry	2
Protocol	24	Where the full trial protocol can be accessed, if available	5
Funding	25	Sources of funding and other support (such as supply of drugs), role of funders	15

*We strongly recommend reading this statement in conjunction with the CONSORT 2010 Explanation and Elaboration for important clarifications on all the items. If relevant, we also recommend reading CONSORT extensions for cluster randomised trials, non-inferiority and equivalence trials, non-pharmacological treatments, herbal interventions, and pragmatic trials. Additional extensions are forthcoming: for those and for up to date references relevant to this checklist, see www.consort-statement.org.
